# In vivo toxicity assessment of silica-coated iron oxide nanoparticles for nanowarming in organ cryopreservation

**DOI:** 10.1186/s12951-026-04471-6

**Published:** 2026-05-07

**Authors:** Onyinyechukwu Justina Oziri, Cameron Scheithauer, Henry L. Wong, Michael L. Etheridge, Erik B. Finger, John C. Bischof

**Affiliations:** 1 Department of Mechanical Engineering, University of Minnesota, Minneapolis, MN USA; 2 Institute for Therapeutics Discovery and Development, University of Minnesota, Minneapolis, MN USA; 3 Department of Surgery, University of Minnesota, Minneapolis, MN USA; 4 Department of Biomedical Engineering, University of Minnesota, Minneapolis, MN USA; 5 Institute for Engineering in Medicine, University of Minnesota, Minneapolis, MN USA

**Keywords:** Silica coated iron oxide nanoparticles, Nanowarming, Toxicity, Cryopreservation, Male sprague-dawley rats

## Abstract

The ability to cryopreserve and rewarm whole organs without damage has the potential to transform transplantation by enabling long-term banking and global organ sharing. Successful transplantation of rat kidneys cryopreserved and rewarmed using nanowarming, a technique in which silica coated iron oxide nanoparticles (sIONP) are perfused throughout the organ’s vasculature along with cryoprotective agents (CPAs), which are then both washed out of the organ after rewarming prior to transplant has recently been reported. To support development towards clinical organ scale translation, the potential toxicity of sIONP in male Sprague-Dawley rats was evaluated, administering doses six to seven orders of magnitude higher than the estimated systemic exposure corresponding to the highest reported residual iron levels in organs following nanowarming and cryopreservation (3.36 ng Fe/kg). These doses are effectively within the range of a theoretical worst-case scenario, where a fully loaded kidney is transplanted (10.5–14 mg Fe/kg) without washout. Toxicity was assessed at 24 h for (5, 12, and 20 mg Fe/kg) and over 28 days for 12 mg Fe/kg dose. Furthermore, plasma pharmacokinetics, hematology, clinical chemistry, biodistribution and histopathology were evaluated. No overt toxicity was observed at the lower doses, while some adverse effects emerged at the highest dose of 20 mg Fe/kg. These findings suggest minimal toxicity associated with sIONP injections of a dose equivalent to a fully loaded organ, supporting the translational potential of sIONP for organ nanowarming.

**Graphical Abstract:**

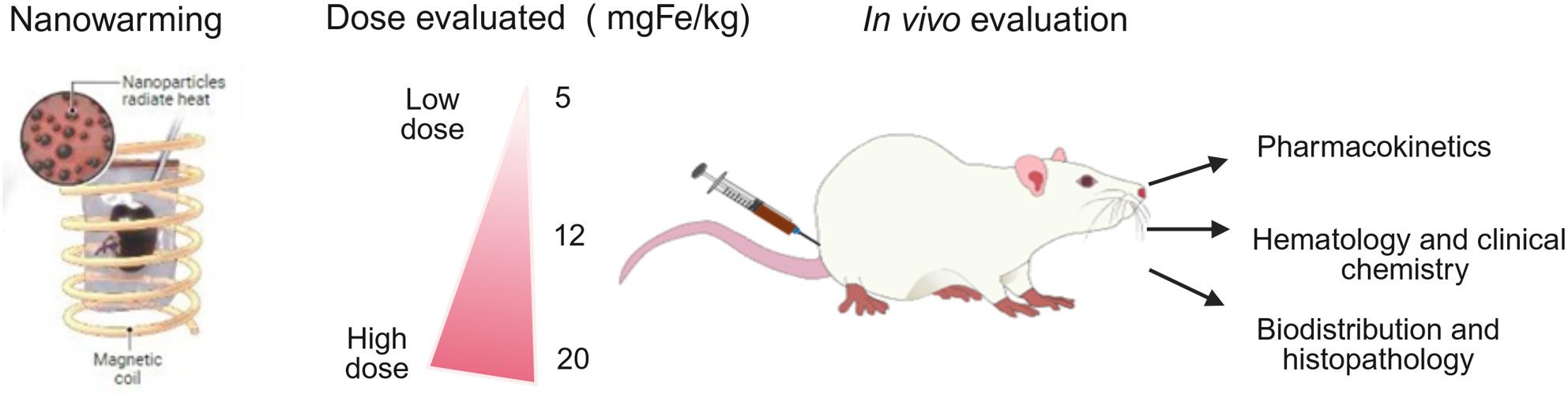

**Supplementary Information:**

The online version contains supplementary material available at 10.1186/s12951-026-04471-6.

## Introduction

In recent years, the global burden of organ-related diseases and end-stage organ failure has continued to climb, driving mortality rates ever higher. Organ transplantation remains a cornerstone therapy for patients with terminal organ dysfunction but persistent challenges; such as inability to use some available organs due to logistical bottlenecks, stem largely from the narrow time window for clinical use of donated organs (typically 4–24 h) [1–3]. Cryopreservation offers a compelling solution by decoupling donor-recipient timing constraints through the extended storage of organs [2]. For instance, successful cryopreservation of a rat kidney for up to 100 days has been reported, followed by nanowarming and transplantation into a nephrectomized rat, where it fully sustained the recipient’s life [4]. Furthermore, physical studies have demonstrated that nanowarming can be applied at the scale of human organs [5]. This suggests that cryopreservation via nanowarming has the potential to address many logistical challenges in transplant medicine, thereby improving patient outcomes and enabling the establishment of organ banks. However, several developmental challenges remain before clinical implementation can be realized. In this work, we address a key translational question by evaluating the toxicity profile of the nanoparticles used in nanowarming.

**Fig. 1:**
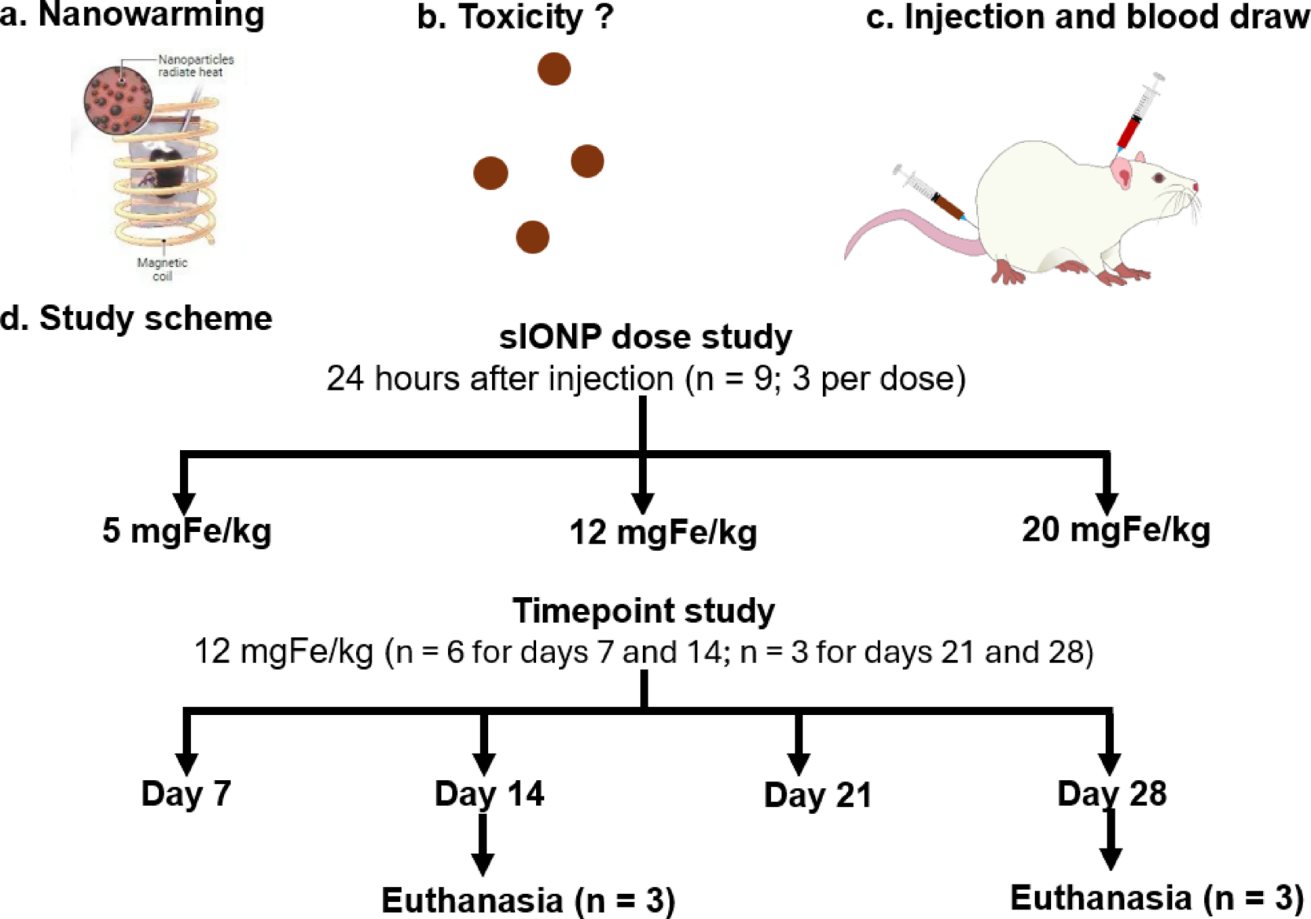
Schematic representation of silica coated iron oxide (sIONP) in nanowarming and in vivo study scheme: (**a**) sIONP loaded and vitrified rat kidney in a magnetic coil; (**b**) sIONP with question of toxicity; (**c**) Rat showing intravenous injection of sIONP via femoral vein and blood collection via jugular vein; (**d**) Study scheme for in vivo assessment of sIONP (*n* = 9; 3 per dose, and *n* = 6 for days 7 and 14; *n* = 3 for days 21 and 28). Vehicle control for dose (*n* = 3) and timepoint (*n* = 3, euthanized on day 28). Figure 1a was taken with permission from Science [16]

Nanowarming is a scalable, volumetric heating method that uses iron oxide nanoparticles (IONP) (Fig. 1) and has shown remarkable effectiveness in rewarming vitrified organs. Vitrified cryopreservation depends on extremely rapid cooling and rewarming to prevent ice formation, allowing tissues to reach and maintain a stable, glass-like state at cryogenic temperatures with the help of cryoprotective agents. Before the development of nanowarming, this technique was constrained by the inability to reheat organs quickly and uniformly enough, which often led to cracking or ice-induced damage. In nanowarming, IONP are dispersed in a colloidal solution and delivered with CPAs throughout the organ’s vasculature and surrounding tissues prior to vitrification [4, 6–10]. Importantly, the IONP are sized to remain in the vasculature, achieving a uniform distribution across capillary beds while allowing ready washout during perfusion unloading. Upon CPA and IONP loading, these organs are vitrified, stored, and can be rewarmed upon demand by applying an alternating magnetic field generated in a radiofrequency coil, thereby heating the IONP within the vasculature and therefore the organ. This enables rapid and sufficiently uniform rewarming throughout the organ, thereby preventing thermal gradients and reducing the risk of ice crystal formation or cracking respectively. The key to successful nanowarming is the stability, heating efficiency, and biocompatibility of the IONP [6, 11, 12]. For instance, silica coated IONP (sIONP) maintain stability in CPAs, efficient heating, can be produced and purified in a scalable manner and show biocompatibility as assessed in human dermal fibroblast cells (HDF), blood vessels, and whole organs [6, 12–14].

After rewarming, a washout step is used to remove the sIONP and CPA prior to transplantation and organ assessments [4, 8–10, 15]. Residual iron has been reported in cryopreserved and nanowarmed organs following washout (Table 1), with the highest level measured at 2.40 µg Fe/mg dry tissue (3.36 ng Fe/kg body weight) [9]. In recent reported work, cryopreserved kidneys that supported successful transplants contained only 1.23 ± 0.78 µg Fe/mg dry tissue, thanks to improvements in synthesis and purification of IONP with Tangential Flow Filtration (TFF) that also allowing scaling while maintaining colloidal stability in CPA [13]. This translates to an estimated systemic exposure of 1.75 ng Fe/kg body weight [13].

To illustrate a theoretical worst-case scenario, transplantation of a kidney fully loaded with nanoparticles was considered. Assuming a vascular volume of 15–20%, a kidney weight of 3.68 g, and a perfusion concentration of 10 mg Fe/mL (Table 1), such a kidney would contain 10.5–14 mg Fe/kg of rat body weight. Although transplanting a fully loaded kidney is impractical and unlikely, this upper bound informed our selection of appropriate dosing concentrations.

As nanowarming advances toward potential clinical use, it is critical to establish the in vivo biosafety profile of the sIONP. This study was conducted to assess this safety by injecting sIONP intravenously into rats at equivalent iron doses six to seven orders of magnitude higher than would occur with the highest reported residual iron in organs (Table 1). This assumes that all residual iron is released systemically at the time of transplant, which is, again, a theoretical worst-case scenario. Animals at 24 h post-injection for the 5, 12, and 20 mg Fe/kg dosages, and after 28 days post-injection for the 12 mg Fe/kg dosage were assessed (Fig. 1). Pharmacokinetics (volume of distribution, clearance, and half-life), hematology (hemoglobin (Hb), hematocrit (HCT), mean corpuscular volume (MCV), mean corpuscular hemoglobin (MCH), mean corpuscular hemoglobin concentration (MCHC), platelets (PLT), white blood cells (WBC), red blood cells (RBC), percenatge neutrophil, absolute neutrophil count (ANC), percentage lymphocyte, and absolute lymphocyte count (ALC)), and blood chemistry parameters were evaluated. This included markers of liver and kidney functions such as blood urea nitrogen (BUN), creatinine, total protein, albumin, alanine aminotransferase (ALT), aspartate aminotransferase (AST), total bilirubin, and alkaline phosphatase (ALP), electrolytes (sodium, chloride, phosphorus, bicarbonate, potassium, and calcium). In addition, glucose, body-weight changes, biodistribution and histopathology of the heart, liver, kidneys, and spleen were assessed.

## Results and discussion

### Overview of IONP in nanowarming

Various formulations of iron oxide nanoparticles (IONP) have been employed for nanowarming and cryopreservation of different biological systems, including cells, tissues, and whole organs [9–13, 15, 17–19]. While these applications have demonstrated promising results, clinical organ scale translation of nanowarming requires two critical benchmarks to be met: (1) effective removal of IONP from the biological system during the washout phase, and (2) demonstration of in vivo biosafety of residual IONP. The extent of residual iron in organ systems following nanowarming and subsequent washout is an important safety consideration, and is impacted by the IONP functionalization and purification [13]. A summary of reported residual iron levels across different studies and organs is provided in Table 1.

**Table 1:** Summary of reported residual iron levels and estimations across studies and organs

Organ	Nanoparticles	Protocol	Perfusion concentration (mg Fe/mL)	Residual iron in the organ (µgFe / dry weight)	Estimated residual iron in the organ (µgFe /wet weight)	Estimated systemic dosing (ngFe/kg)	Animal body weight (g)	Organ assessment	References
Kidney	sIONP	PLU	10	2.00	0.40	2.80	200–250	MRI and ICP-OES	[12]
Kidney	sIONP	PLU, VR	10	2.40	0.48	3.36	176–300	Histology and Confocal imaging	[9]
Kidney	sIONP	PLU, VR, TX	10	1.23	0.25	1.75	450–525	Histology, ICP-MS and transplantation	[13]
Heart	sIONP	PLU, VR	10	1.20	0.24	1.20	250–300	Histology	[8]
*Heart	SPION	PLU, VR	5	10.2		31.0	300	Magnetic particle imaging	[15]

PLU: Perfusion load and unload, VR: Vitrification and rewarming, TX: Transplantation

* Reported values extracted from [15]. Calculations shown in Table S1

Gao et al. reported the first quantitative assessment of iron residuals following scalable silica-coated iron oxide nanoparticle (sIONP) perfusion and washout in rat kidneys, with a measured concentration of 2.0 µg Fe/mg kidney dry weight [12]. However, vitrification and nanowarming were not performed prior to the washout. Sharma et al. implemented a full cryopreservation protocol comprising CPA/sIONP loading, vitrification, nanowarming, and washout and reported a slightly higher residual iron level of 2.4 µg Fe/mg kidney dry weight [9]. More recently, the perfusion loading/unloading, vitrification, and rewarming protocols were optimized and, importantly, continued to develop the sIONP purification process using TFF over ultracentrifugation [4, 20]. This included comprehensive biological assessments, achieving 1.23 ± 0.78 µg Fe/mg kidney dry weight and demonstrating successful post-operative survival following transplantation [13]. In cardiac tissues, Chiu-Lam et al. reported 10.2 ± 2.7 µg Fe residual iron after perfusion, cryopreservation, nanowarming, and wash-out of whole rat heart with PEG-silane coated superparamagnetic iron oxide nanoparticles [15], while Gao et al. vitrified and nanowarmed rat hearts using sIONP and found iron residuals of 1.2 µg Fe/mg dry weight post-washout [8].

Several additional studies have demonstrated the effectiveness of organ vitrification and nanowarming, although they do not provide data on iron residual levels. A few examples include Zhan et al. enhanced structural preservation of vitrified rat kidneys using carboxylic-acid functionalized iron oxide nanoparticles [21], and Sharma et al. performed sIONP-based nanowarming of cryopreserved rat livers, yet neither reported post-washout iron levels [9]. In a key advancement, Han et al. achieved the first life-sustaining rat kidney transplantation following vitrification and nanowarming, but also did not report post-washout iron levels [4]. Across all published work, the highest documented residual iron in a cryopreserved and rewarmed organ is 2.4 µgFe/mg dry kidney tissue corresponding to an estimated systemic exposure of approximately ~ 3.36 ng Fe/kg [9]. Establishing in vivo biosafety at doses equivalent to or exceeding these residual levels is therefore critical for clinical translation of organ-scale nanowarming.

To address this, an in vivo toxicity study in rats (6–8 weeks old) was conducted, administering sIONP intravenously at 5, 12, or 20 mg Fe/kg, which are orders of magnitude above the highest document residual iron content in nanowarmed tissues. All animals survived a 24-hour post-injection period, after which they were euthanized for acute assessment, while a second group received intravenous injection of sIONP at dose of 12 mg Fe/kg for extended evaluation over 28 days (Fig. 1). Blood samples were drawn via the jugular catheter for plasma pharmacokinetics, clinical chemistry, and hematology. Biodistribution and histopathology analyses were performed on liver, kidney, spleen, and heart 24 h, day 14, and day 28 after injection.

### sIONP characterization and preparation for in vivo use

sIONP were synthesized using previously published protocols [12, 13]. Prior to in vivo administration, the physicochemical properties of the synthesized and purified sIONP were characterized. Dynamic light scattering (DLS) (Fig. S1a) indicated a hydrodynamic diameter of 86 ± 2 nm, and ζ-potential measurements revealed a strongly negative surface charge of − 41 ± 3 mV. Transmission electron microscopy (TEM) confirmed sIONP size of 50 ± 2 nm (Fig. S1b). These results are in line with expected results based on the established synthesis methods [12, 13].

### Plasma pharmacokinetics

Understanding plasma pharmacokinetics is essential for optimizing nanoparticle design, surface modification, and overall safety during translation for clinical scale organs. In this study, three key pharmacokinetic parameters: the volume of distribution (Vd _sIONP_) which measures the apparent volume into which the sIONP appears to be distributed after intravenous administration; plasma clearance of sIONP (CL _sIONP_), which is the rate at which sIONP is removed from the bloodstream; and half-life (T_1/2 sIONP_ ), the time required for plasma concentration of sIONP to reach half of its initial value were evaluated. Together, these metrics reveal how rapidly sIONP clear from circulation and distribute into tissues.

**Fig. 2:**
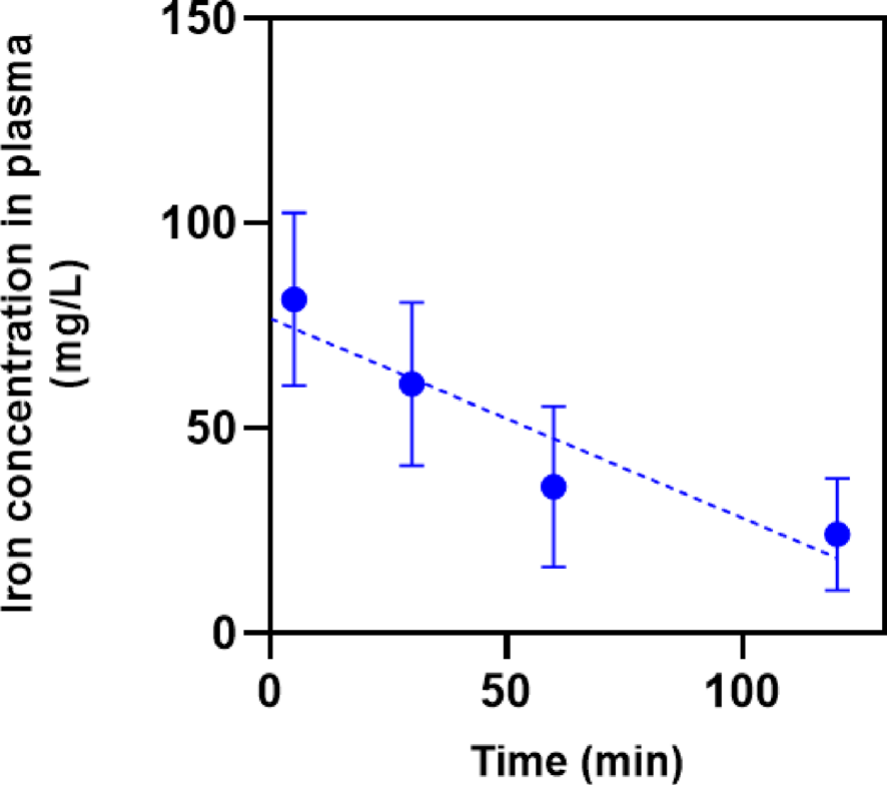
Plasma iron concentration over 120 min following intravenous injection of 12 mg Fe kg⁻¹ sIONP in rats. Plasma iron levels (mg L⁻¹) in *n* = 3 rats

Rats received intravenous injections of sIONP at a dose of 12 mg Fe/kg. Plasma samples were collected at 5, 30, 60, and 120 min post-injection, digested, and iron concentrations in the plasma were measured using ICP-MS. These data were used to calculate pharmacokinetic parameters. Figure 2 shows plasma iron levels over time following injection. A peak plasma iron level was observed at the earliest time point assessed (5 min), followed by a progressive decline. This decrease is likely attributable to sequestration by the mononuclear phagocyte system (MPS), particularly within the liver and spleen.

The volume of distribution (V_d_) of sIONP was 32.0 ± 1.0 mL (158.0 ± 7.0 mL/kg when normalized by body weight), and was calculated individually for each rat using the following equation:

$$\:{{\mathrm{V}}_{\mathrm{d}}}_{\:}\left(\mathrm{L}\right)=\frac{\mathrm{D}\mathrm{o}\mathrm{s}\mathrm{e}\:\left(\mathrm{m}\mathrm{g}\right)}{{\mathrm{C}}_{0}\:\left(\mathrm{m}\mathrm{g}/\mathrm{L}\right)}$$

where:

V_d_ = volume of distribution (L)

Dose = dose of sIONP administered (mg)

C_0_ = initial plasma iron concentration (mg/L)

sIONP exhibited distribution kinetics consistent with a straight-line graph profile on the plasma iron concentration vs. time curves shown in Fig. 2. Because the initial plasma iron concentration at time zero (C₀) is difficult to measure directly, it was estimated by extrapolating the plasma concentration-time curve back to time = 0.

Plasma clearance rate (CL) of sIONP was 494.0 ± 21.0 µL/min and was calculated using the following equation for each rat:

$$\:\mathrm{C}\mathrm{L}\:(\mathrm{L}/\mathrm{m}\mathrm{i}\mathrm{n})\:=\:\frac{\mathrm{D}\mathrm{o}\mathrm{s}\mathrm{e}\:\left(\mathrm{m}\mathrm{g}\right)}{\mathrm{A}\mathrm{U}\mathrm{C}\:(\mathrm{m}\mathrm{g}/\mathrm{L}\cdot \mathrm{m}\mathrm{i}\mathrm{n})}$$

where:

CL = plasma clearance rate (L/min)

Dose = dose of sIONP (mg)

AUC = area under the plasma concentration time curve (mg/L. min)

Half-life (t_1/2_) of sIONP was 47.0 ± 2.0 min and was calculated for each rat as:

$$\:{{T}_{1}}_{/2}\left(\mathrm{m}\mathrm{i}\mathrm{n}\mathrm{u}\mathrm{t}\mathrm{e}\mathrm{s}\right)=\:\frac{0.693\:\times\:{\mathrm{V}}_{\mathrm{d}}\:\left(\mathrm{L}\right)}{\mathrm{C}\mathrm{L}\:(\mathrm{L}/\mathrm{m}\mathrm{i}\mathrm{n})}$$

where:

T_1/2_ = half-life of sIONP (minutes)

V_d_ = volume of distribution (L)

CL = plasma clearance rate (L/min)

By 24 h post-injection, plasma iron levels in sIONP-treated rats were not statistically different than vehicle control (*p* = 0.487; Figure S2), indicating clearance of sIONP from the bloodstream within 24 h. Compared with other iron oxide nanoparticles used in MRI, sIONP exhibited an intermediate half-life; longer than AMI-25 (6 min) [22], VSOP-C43 (8.4 min) [23], VSOP-C184 (21 min) [24], and SHU 555 C (Resovist, 35 min) [25]; but shorter than Ferumfoxtrane-10 (Sinerem, 81 min) [22, 26], USPIO (2 h) [27, 28], Ferumoxytel (AMI17220, 67 min) [25], SPIO (90 min) [29], USPIO sinerem (4 h 30 min) [30], and BMS 1,805,491 (USPIO, 3.7 h) [31]. It is important to note that IONP pharmacokinetics also depends strongly on dose [32], particle size [33, 34], core morphology [35], coating chemistry [36–41], surface charge and zeta potential [42, 43], as well as the animal’s age [23], strain, and immune status [44].

### Hematology and clinical chemistry

Evaluating changes in standard hematological and clinical chemistry parameters after intravenous sIONP administration is essential for assessing systemic safety. Our synthesized sIONP have a core size of ~ 10 nm and an 86 ± 2 nm hydrodynamic diameter, dimensions comparable to viruses or proteins that can interact with the immune system and alter blood-cell parameters or trigger inflammation [45–47]. Nanoparticle fate in circulation is strongly influenced by size [48, 49], surface chemistry [50, 51], coating [52, 53], and dose [54], which collectively govern interactions with blood components and subsequent clearance or degradation.

**Fig. 3:**
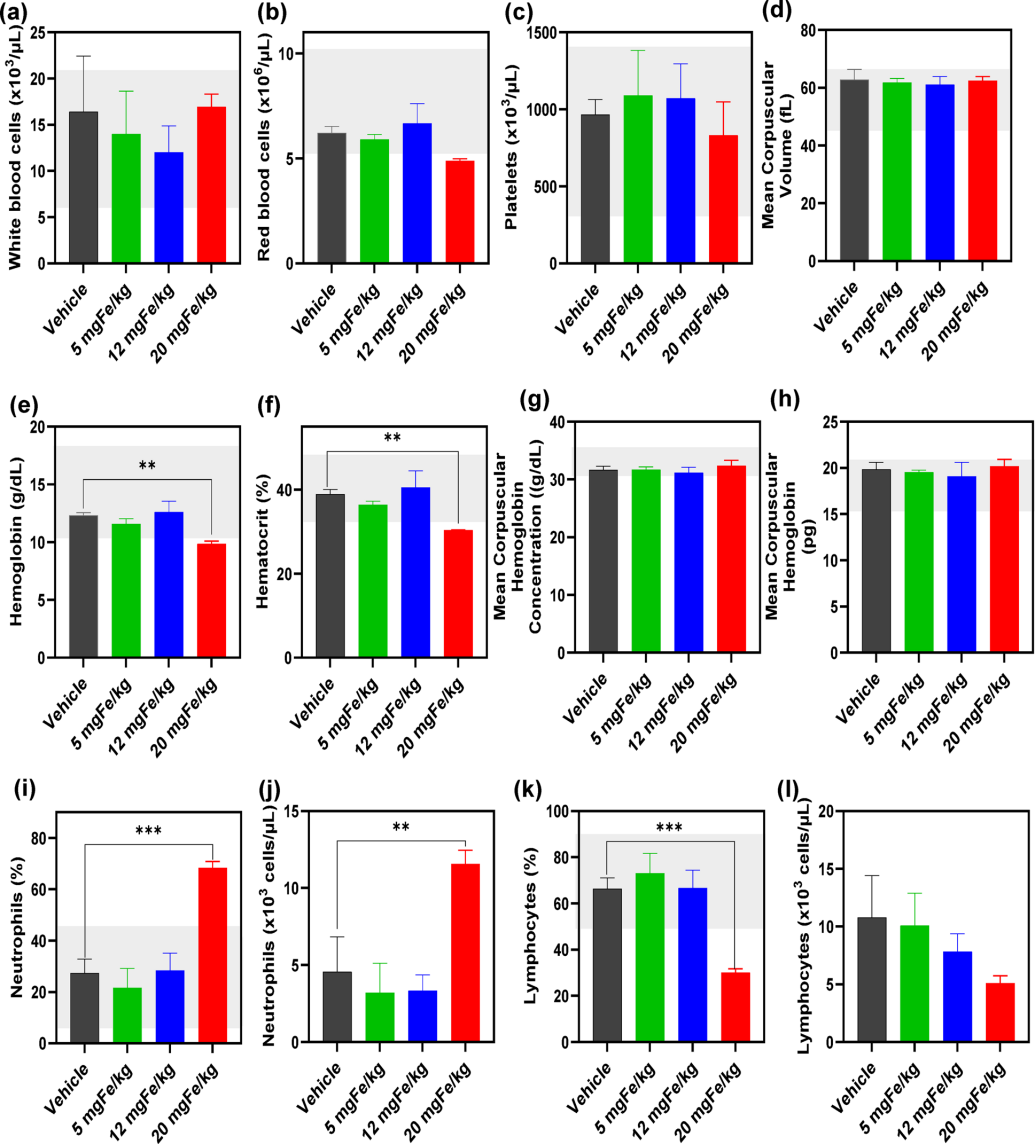
Hematological profiles in rats 24 h after 5, 12, or 20 mg Fe/kg of sIONP injection. Data represents the mean ± standard deviation for the units as shown for (**a**) white blood cells, (**b**) red blood cells, (**c**) platelets, (**d**) mean corpuscular volume, (**e**) hemoglobin, (**f**) hematocrit, (**g**) mean corpuscular hemoglobin concentration, (**h**) mean corpuscular hemoglobin, (**i**) percentage neutrophil, (**j**) absolute neutrophil count, (**k**) percentage lymphocytes, and (**l**) absolute lymphocyte count. Grey bars indicate the normal range for healthy rats, based on previously reported values [55, 56]. Statistical comparison between vehicle control and treatment groups (5, 12, or 20 mg Fe/kg) were performed using one-way ANOVA followed by Tukey’s post hoc test with *n* = 3 per group. **p ˂ 0.01 and ***p ˂0.001. Full p-values are provided in Table S2

Over a 24 h period, hematology parameters in rats after intravenous injection of control, 5, 12 or 20 mg Fe/kg dose (Fig. 3) were evaluated. All animals survived the 24 h post-injection period. Standard hematological markers; Hb, HCT, MCV, MCH, MCHC, PLT, WBC, RBC, percentage neutrophil, and percentage lymphocyte remained within the normal physiological established reference ranges of rat for 5 and 12 mg Fe/kg groups (Fig. 3), suggesting there was no overt toxicologically relevant effect on hematology markers from sIONP exposure [55, 56].

In contrast, Fig. 3 shows that at a 20 mg Fe/kg dose there was a slight reduction in RBC count (*p* = 0.0514) and statistically significant decreases in Hb (*p* = 0.0030), HCT (*p* = 0.0049), and percentage lymphocytes (*p* = 0.0005), which fall outside the normal range expected for rats [55]. These findings suggest interactions with blood components and additional physiological alterations following exposure. The observed decrease in RBC and Hb (anemia) could reflect hemolysis [57, 58]. The concurrent drop in HCT further suggests reduced blood viscosity, likely driven by lower erythrocyte counts or diminished hemoglobin per cell [59, 60]. Additionally, the ANC (*p* = 0.0034) and percentage neutrophils (*p* = 0.0001) increased by 2.5-fold at 20 mg Fe/kg, which may result from demargination of neutrophils from the vasculature primarily in the lungs or from inflammation or infection. In summary, intravenous sIONP doses of 5 and 12 mg Fe/kg showed no overt hematological toxicity, whereas the 20 mg Fe/kg dose induced anemia and signs of acute innate immune activation. Although mechanistic immune profiling was beyond the scope of this application specific safety study, the observed changes are consistent with expected acute inflammatory responses to a high systemic nanoparticle burden. Future studies can incorporate detailed immunophenotyping to further elucidate the mechanistic basis of these responses.

**Fig. 4:**
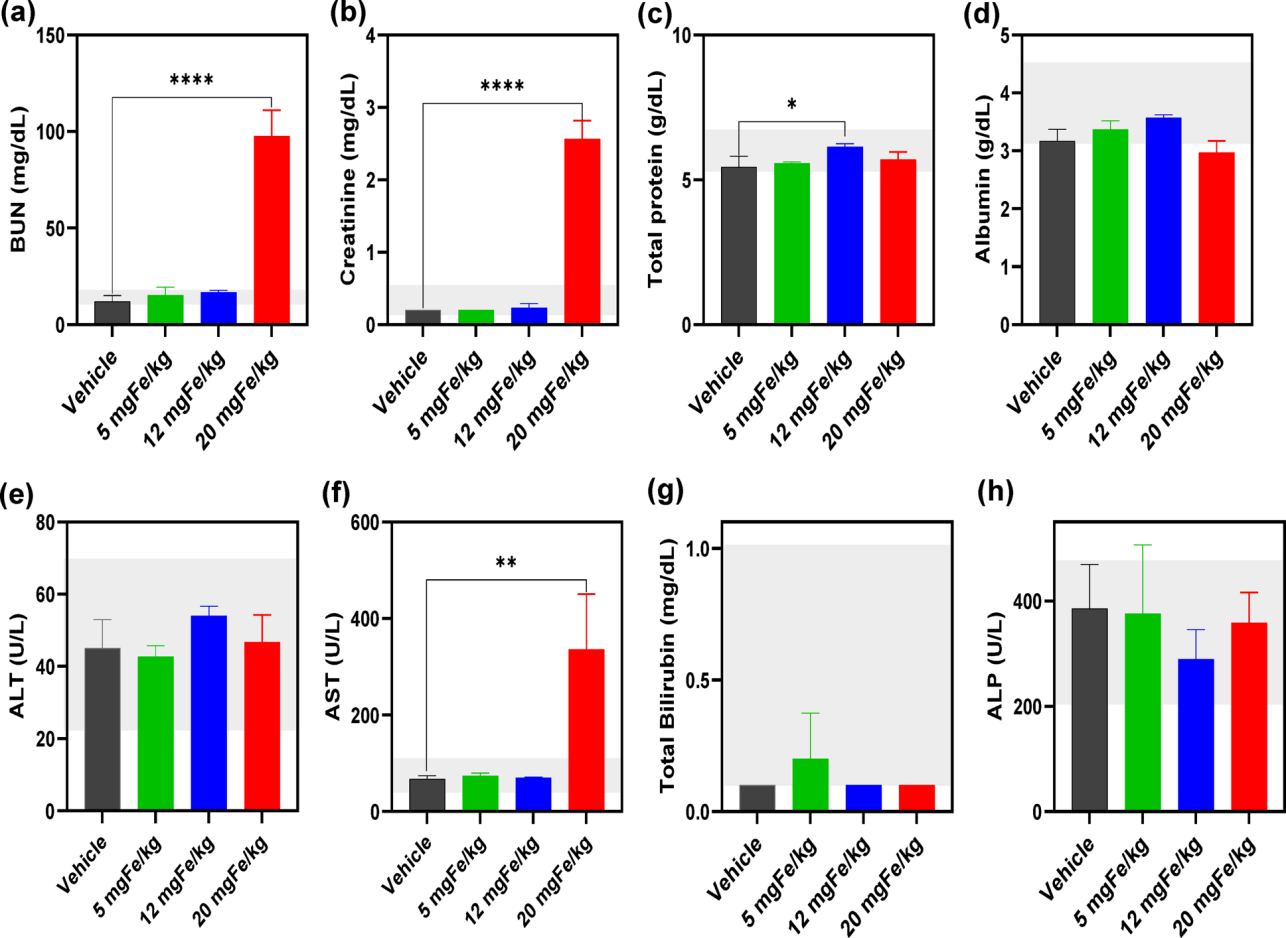
Blood biochemistry in rats 24 h after 5, 12, or 20 mg Fe/kg of sIONP injection. Data represents the mean ± standard deviation of units as shown for (**a**) blood urea nitrogen, (**b**) creatinine, (**c**) total protein, (**d**) albumin, (**e**) alanine aminotransferase, (**f**) aspartate aminotransferase, (**g**) total bilirubin, and (**h**) alkaline phosphatase. Grey bars indicate the normal range for healthy rats, based on literature values [55, 56]. Statistical comparison between vehicle control and each treatment groups (5, 12, or 20 mg Fe/kg) were performed using one-way ANOVA followed by Tukey’s post hoc test (*n* = 3 per group). *p ˂ 0.05, **p ˂ 0.01, and ****p ˂0.0001. Full p-values are provided in Table S2

After contact with blood components, nanoparticles may be transported and accumulate in organs, particularly the liver, kidney, and spleen, where they may be degraded over time. To assess potential organ toxicity, biochemical indicators of kidney and liver function were measured (Fig. 4). Kidney function was evaluated via BUN and creatinine. Liver injury was assessed by measuring ALT, AST, ALP, and total bilirubin. Liver function was assessed by serum albumin and total serum protein levels. At doses of 5 and 12 mg Fe/kg, all kidney and liver biomarkers remained within the physiological range except total protein at 12 mg Fe/kg, which showed a statistically significant difference (*p* = 0.0297) but still remained within the expected range for rats (Fig. 4) [55, 56]. In contrast, the 20 mg Fe/kg group showed an 8-fold increase in BUN (p = ˂ 0.0001) and a 13-fold increase in creatinine (p = ˂ 0.0001), both statistically significant and exceeding the expected physiological range, indicating impaired renal function. Similarly, AST levels were elevated (~ 5-fold, *p* = 0.0020) after 20 mg Fe/kg dosing, and albumin was slightly reduced outside the normal range, consistent with liver injury at this higher dose (Fig. 4). In future studies, additional evaluations of liver and kidney effects would include functional assays (e.g., glomerular filtration rate, urine protein, liver metabolism), organ injury biomarkers (e.g., KIM-1, NGAL, cystatin C, gamma-glutamyl transferase, alkaline phosphatase isoforms, serum bile acids), and molecular analyses of oxidative stress, inflammation, or apoptosis. These complementary approaches would provide a more comprehensive assessment of organ-specific responses and the dose-dependent effects observed.

**Fig. 5:**
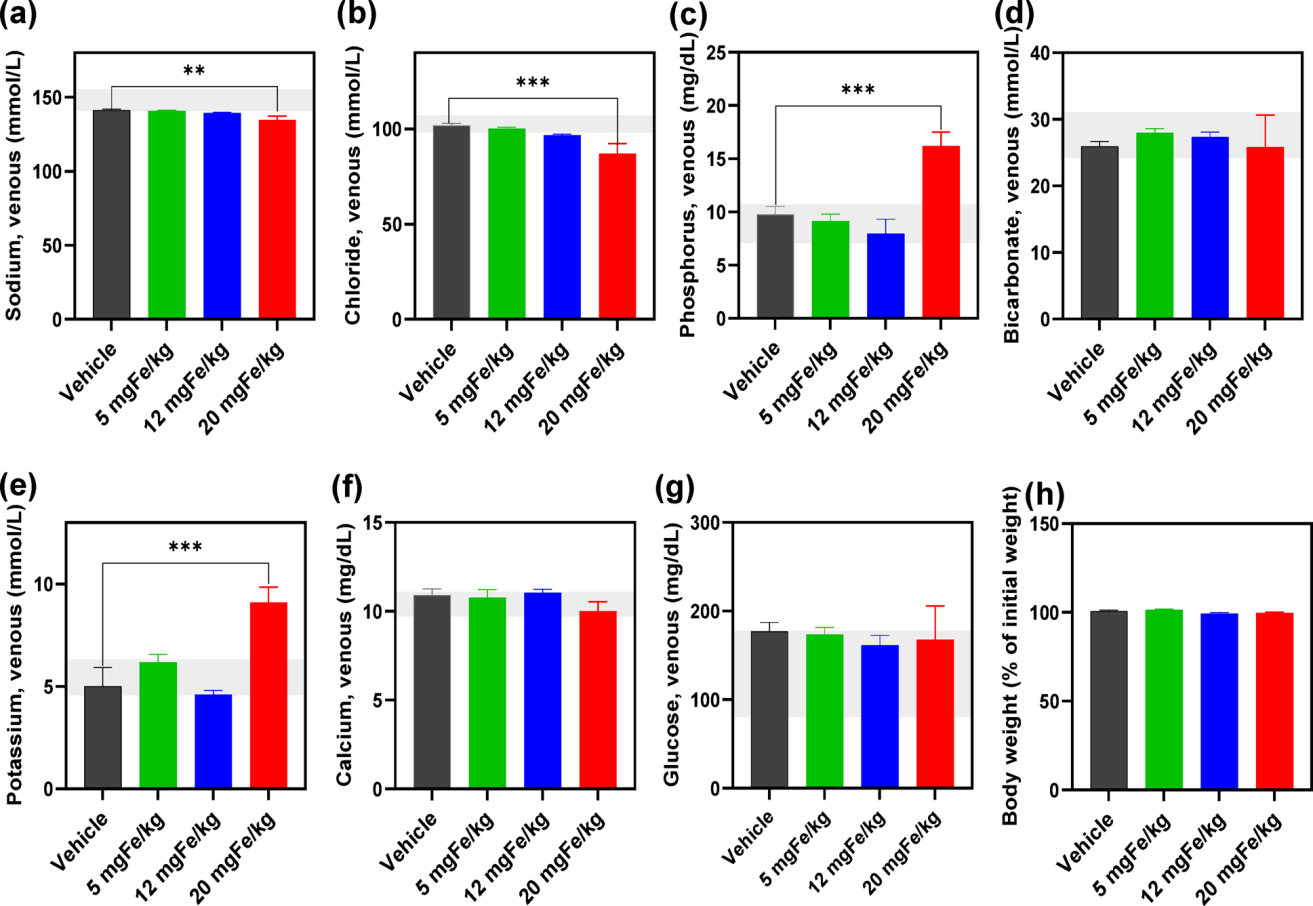
Electrolyte balance, glucose levels, and body weight in rats 24 h after sIONP injection. Concentration of sIONP varied between 5, 12, and 20 mg Fe/kg, or vehicle control. Data represent the mean ± standard deviation of (**a**) sodium, (**b**), chloride, (**c**) phosphorus, (**d**) bicarbonate (**e**) potassium, (**f**) calcium, (**g**) glucose, and (**h**) weight. Grey bars indicate normal ranges for healthy rats [55, 56]. Statistical comparison between vehicle control and each treatment group (5, 12, or 20 mg Fe/kg) were performed using one-way ANOVA followed by Tukey’s post hoc test (*n* = 3 per group). Full p-values are provided in Table S3

Electrolytes (sodium, chloride, phosphorus, bicarbonate, potassium, and calcium) and glucose 24 h after injections at 5, 12, and 20 mg Fe/kg doses (Fig. 5a–g) were measured. At 5 and 12 mg Fe/kg, all values remained within the normal ranges for rats and showed no statistically significant differences (Table S3) [55, 56]. However, in the 20 mg Fe/kg group, several electrolyte disturbances that were statistically significant were observed. Sodium (*p* = 0.0026) and chloride (*p* = 0.0009) levels decreased markedly, whereas potassium (*p* = 0.0002) and phosphorus (*p* = 0.0004) levels increased dramatically. These alterations are consistent with electrolyte imbalances likely secondary to impaired renal function at the 20 mg Fe/kg dose (Table S3). Alternatively, elevated phosphorus levels could be due to bone resorption, albeit unlikely in this rapid timeframe.

Notably, throughout the study, none of the rats, whether dosed with vehicle or sIONP displayed unusual behaviors or signs of distress, and body weights were stable across all groups at 24 h post-injection (Fig. 5h).

Prior studies have shown that superparamagnetic iron oxide nanoparticles initially accumulate mainly in the liver and spleen after administration, leading to excretion or undergoing degradation and gradual release back into the bloodstream in various iron bound forms (e.g., Fe ^2+^( Iron II) and Fe ^3+^ (Iron III) [61–63]. These are later incorporated into erythrocyte hemoglobin, proteins, and iron storage complexes such as ferritin and transferrin. To investigate the longer-term systemic effects of sIONP clearance and turnover, hematology and clinical chemistry parameters in rats over 28 days were evaluated. Blood samples were collected on day 7, 14, 21, and 28 post-injection and assayed for hematological parameters: Hb, HCT, MCV, MCH, MCHC, PLT, WBC, RBC, percentage neutrophil, ANC, percentage lymphocyte, and ALC as well as liver and kidney functions markers (BUN, creatinine, total protein, albumin, ALT, AST, total bilirubin, and ALP).

**Fig. 6:**
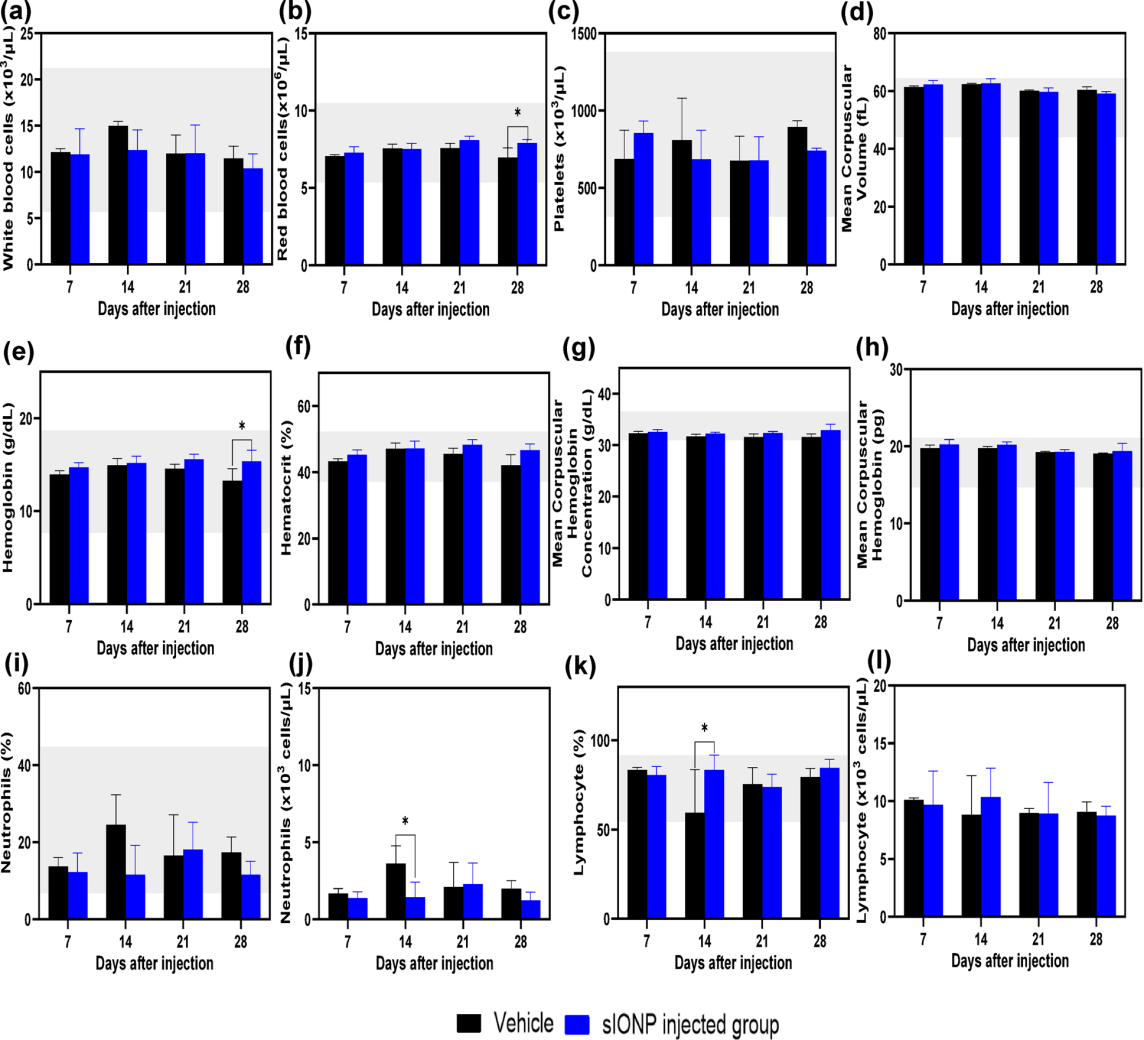
Hematology over time following 12 mg Fe/kg sIONP injection in rats. Measurements were taken 7, 14, 21, and 28 after sIONP injection (12 mg Fe/kg) in rats. Data represents the mean ± standard deviation of the units shown for (**a**) white blood cells, (**b**) red blood cells, (**c**) platelets, (**d**) mean corpuscular volume, (**e**) hemoglobin, (**f**) hematocrit, (**g**) mean corpuscular hemoglobin concentration, (**h**) mean corpuscular hemoglobin, (**i**) percentage neutrophils, (**j**) absolute neutrophil count, (**k**) percentage lymphocytes, and (**l**) absolute lymphocyte count. Grey bars indicate normal ranges based on literature values [55, 56]. Statistical comparison between vehicle control and treatment group were analyzed using multiple *t*-tests, with *p*-values adjusted for multiple comparisons using the Holm–Šidák method (*n* = 6 for days 7 and 14; *n* = 3 for days 21 and 28 or vehicle control). *p ˂ 0.05. Full p-values are provided in Table S4

As shown in Fig. 6, all hematological parameters remained within the established physiological ranges for rats, with no consistent upward or downward trends attributable to sIONP exposure [55, 56]. While statistically significant differences were observed in RBC and hemoglobin on day 28 (*p* = 0.0464 and *p* = 0.0270, respectively), as well as in absolute neutrophil count and percentage lymphocytes on day 14 (*p* = 0.0309 and *p* = 0.0120, respectively), these changes were not associated with values outside normal physiological limits (Table S4). Therefore, although variations were detected, they are unlikely to reflect toxicologically relevant effects over the 28-day study period at the tested dose.

**Fig. 7:**
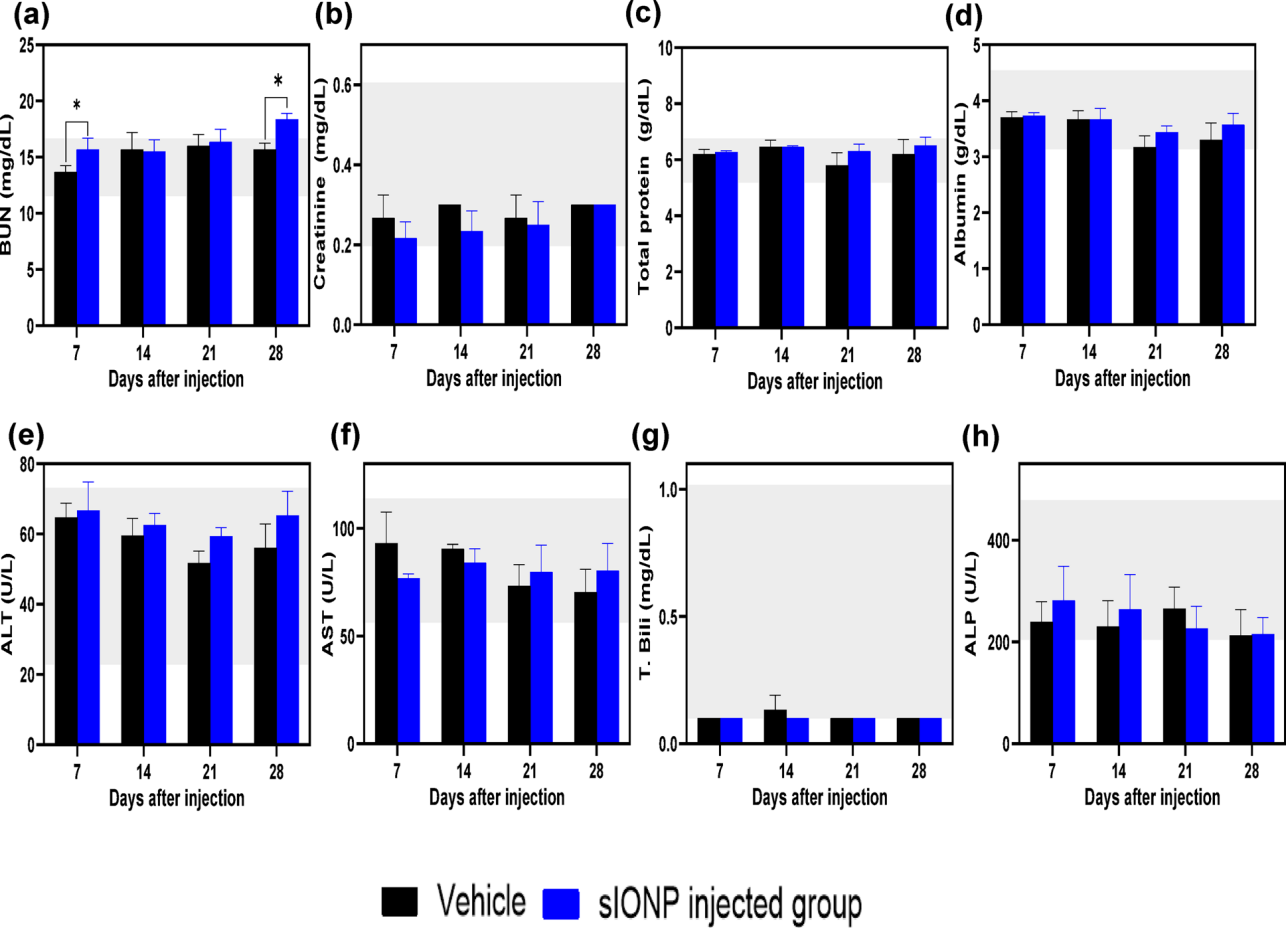
Blood biochemistry levels over time following 12 mg Fe/kg sIONP injection in rats. Serum blood biochemical parameters measured on days 7, 14, 21, and 28 after sIONP injection. Data represent mean ± standard deviation of the units shown for (**a**) blood urea nitrogen, (**b**), creatinine, (**c**) total protein, (**d**) albumin (**e**) alanine aminotransferase, (**f**) aspartate aminotransferase, (**g**) total bilirubin, and (**h**) alkaline phosphatase. Grey bars denote normal reference ranges from healthy rats [55, 56]. Statistical comparison between vehicle control and treatment group at each time point (day 7, 14, 21, or 28) were performed multiple *t*-tests, (**a**,** c**,** d**,** e**,** f**,** h**) or Mann–Whitney *U* (**b**,** g**) with *p*-values adjusted for multiple comparisons using the Holm–Šidák method. *p ˂ 0.05. Injections were into *n* = 6 rats for days 7 and 14; *n* = 3 for days 21 and 28 with *n* = 3 vehicle controls. Full p-values are provided in Table S5

After exiting the bloodstream, nanoparticles have been reported to mainly accumulate and degrade over time in the liver, spleen, and kidneys, depending on their sizes [63–65], so it is essential to monitor liver and kidney biomarkers as indicators of potential toxicity even after acute exposure, as shown in Fig. 7. Rats received a 12 mg Fe/kg dose of sIONP, and blood samples were collected on day 7, 14, 21, and 28 post-injection, and biomarkers indicative of liver and kidney functions such as BUN, creatinine, total protein, albumin, ALT, AST, total bilirubin, and ALP were evaluated. Figure 7 shows that values obtained from sIONP-injected rats were within the normal physiological range for rats, except for an increase in BUN above the expected range on day 28. No significant statistical difference was observed between the 12 mg Fe/kg and vehicle injected rats on days 7, 14, 21, and 28 for total protein, albumin, AST, ALT, total bilirubin, and ALP. Biochemical biomarkers were compared across different time points relative to the vehicle control group. Statistically significant differences were obtained for BUN on days 7 (*p* = 0.0297) and 28 (*p* = 0.0145) which fell outside of the expected physiological range for rats (Fig. 7, Table S5).

**Fig. 8:**
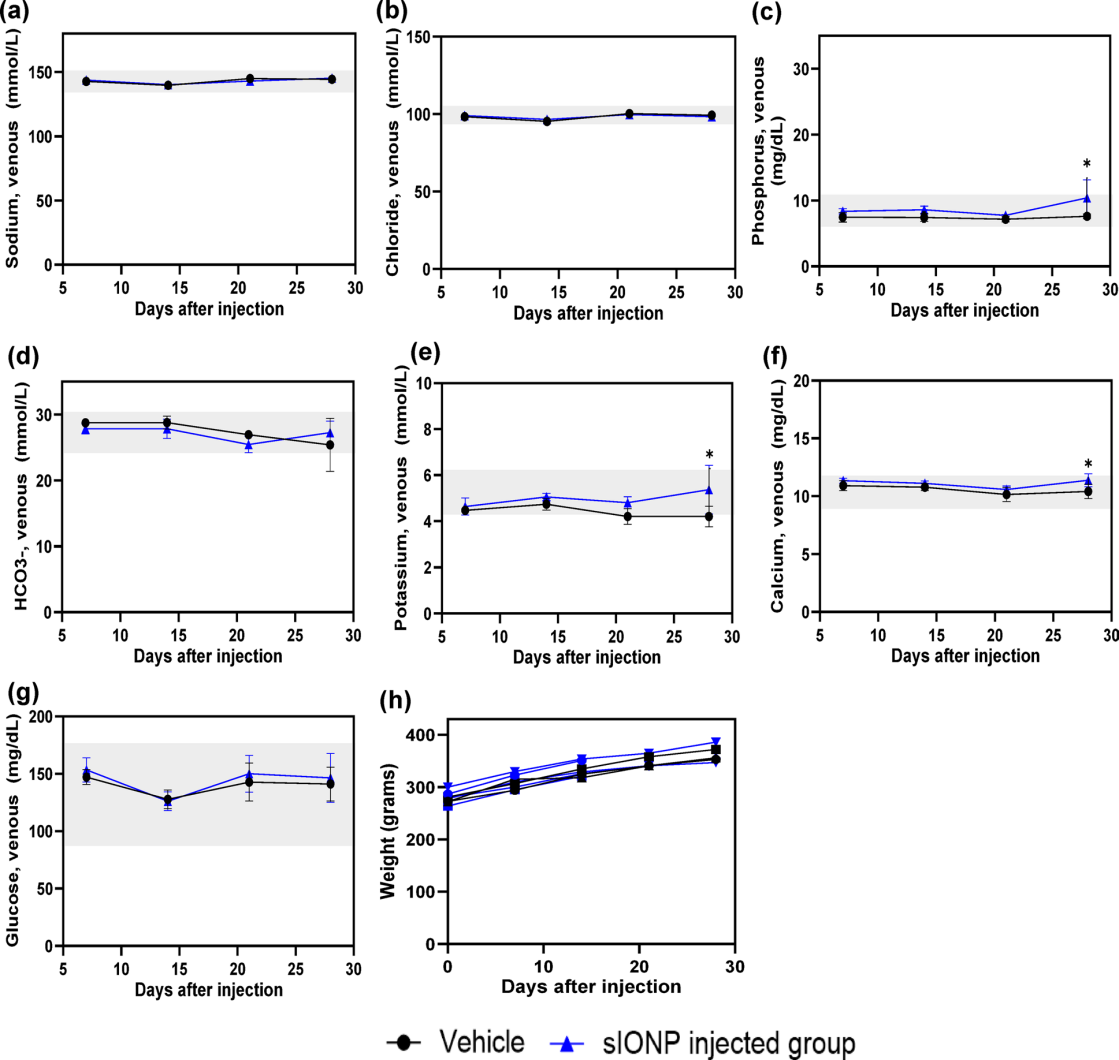
Electrolytes balance, glucose levels, and body weight over time following 12 mg Fe/kg sIONP injection in rats. Electrolyte concentrations and body weight measured on days 7, 14, 21, and 28 after sIONP injection. Data represents the mean **±** standard deviation in the units shown for (**a**) sodium, (**b**), chloride, (**c**) phosphorus, (**d**) bicarbonate (**e**) potassium, (**f**) calcium, (**g**) glucose, and (**h**) body weight. Grey bars indicate reference ranges for healthy rats, based on published values [55, 56]. Statistical comparison between vehicle control and treatment group at timepoints (day 7, 14, 21, or 28) were performed using multiple *t*-tests, with *p*-values adjusted for multiple comparisons using the Holm–Šidák method. *p ˂ 0.05. Injections were into *n* = 6 rats for days 7 and 14; *n* = 3 for days 21 and 28 or vehicle control. Full p-values are provided in Table S6

Electrolyte (sodium, chloride, phosphorus, bicarbonate, potassium, and calcium) and glucose levels, measured at days 7, 14, 21, and 28 following a 12 mg Fe/kg sIONP injection, remained within established physiological ranges for rats [55, 56] (Fig. 8a–g). Statistically significant differences were observed in phosphorus, potassium, and calcium on day 28 (*p* = 0.0061, *p* = 0.0114, and *p* = 0.0249, respectively; Table S6). However, all values remained within normal limits. Body weights increased steadily over the 28-day period (Fig. 8h), and no animals exhibited abnormal behavior or signs of distress at any time point.

### Biodistribution of sIONP in organs

Biodistribution is a critical factor in evaluating toxicity. In this study, sIONP at multiple doses were administered to assess their distribution in vivo in rats. The mononuclear phagocytic system (MPS), such as the liver and spleen, are well established accumulation sites of nanoparticles after administration into the body and contact with the blood stream [66–68]. Typically, Kupffer cells in the liver sinusoids and splenic red pulp macrophages take up nanoparticles by endocytosis, digest and degrade them in the lysosomes. Elimination from the body is carried out by additional metabolic pathways, leading to excretion in urine or feces [69]. Herein, biodistribution of sIONP in the liver, kidney, spleen, and heart 24 h after injection and a 28-day period were evaluated.

**Fig. 9:**
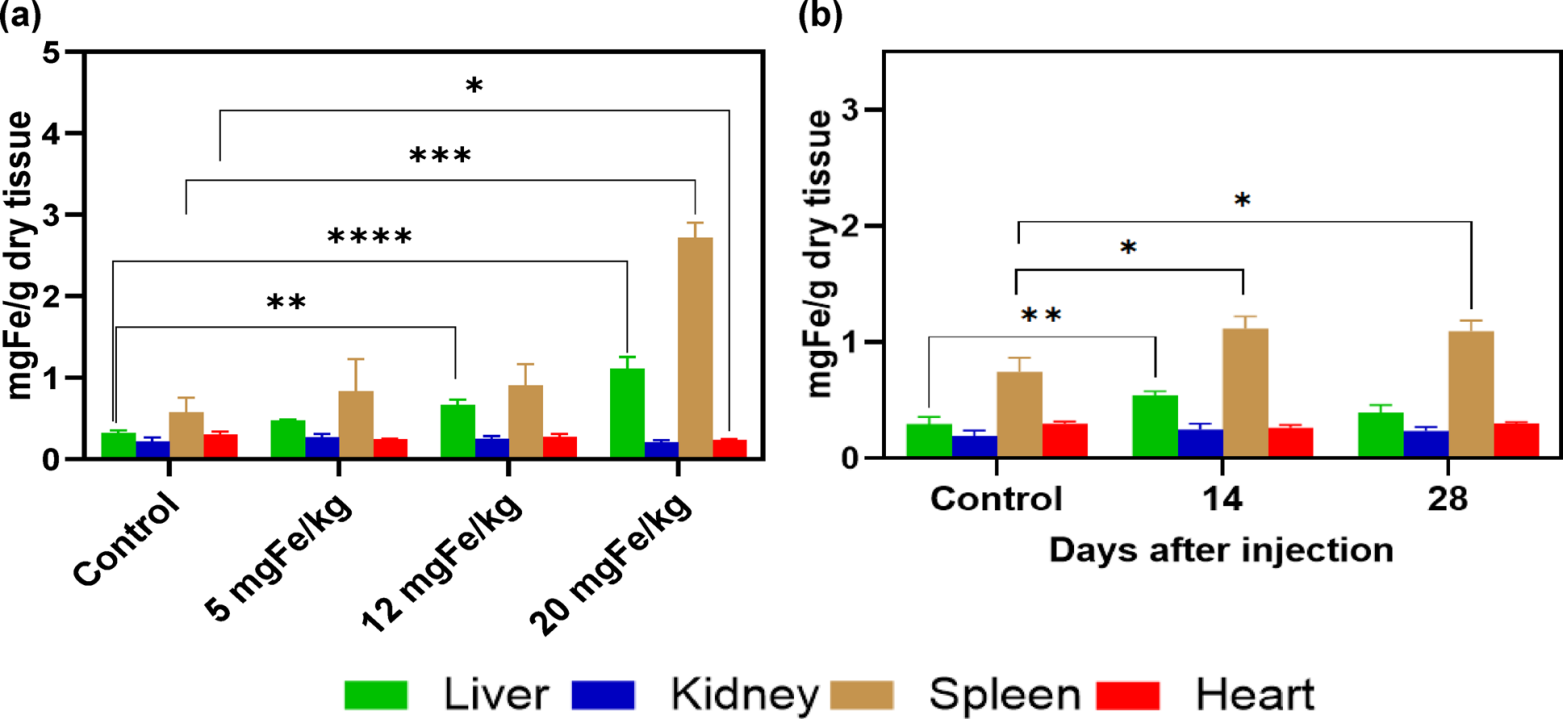
Biodistribution of sIONP in major organs following intravenous administration. (**a**) Organ-specific iron levels 24 h post-injection of sIONP at 5, 12 or 20 mg Fe/kg, (**b**) Organ-specific iron levels at 14 and 28 days after injection of 12 mg Fe/kg sIONP. Data represent mean ± standard deviation. Statistical comparison between vehicle control and each treatment groups (5, 12 or 20 mg Fe/kg, day 14 or 28) were performed using one-way ANOVA followed by Tukey’s post hoc test with *n* = 3 per dose. A two-tailed unpaired *t*-test was used for timepoint comparisons. *p ˂ 0.05, **p ˂ 0.01, ***p ˂0.001, and ****p ˂0.0001. Full p-values are provided in Table S7 and S8

As shown in Fig. 9a, liver iron levels increased significantly in a dose-dependent manner, from 0.322 ± 0.035 mg Fe/g dry tissue in control to 0.671 ± 0.061 mg Fe/g (12 mg Fe/kg dose; *p* = 0.0034) and 1.108 ± 0.146 mg Fe/g (20 mg Fe/kg dose; p = ˂ 0.0001). Similarly, spleen iron levels rose from 0.576 ± 0.183 mg Fe/g in controls to 2.720 ± 0.184 mg Fe/g following the 20 mg Fe/kg dose (*p* = 0.0003). In contrast, kidney iron levels showed no statistically significant changes across the groups. However, although overall iron level was low in the heart, they were significantly reduced from 0.303 ± 0.037 mg Fe/g in controls to 0.236 ± 0.012 mg Fe/g at 20 mg Fe/kg (*p* = 0.0431) (Fig. 9a, Table S7).

Our data clearly demonstrate dose-dependent uptake by the MPS, with minimal renal involvement. The reduction in the heart iron levels may be attributed to the level of expressed transferrin receptors, the binding of iron to proteins or the redistribution of iron to other tissues [70]. Also, the high iron accumulation observed in the liver and spleen at 12 and 20 mg Fe/kg doses suggests rapid recognition by Kupffer cells of the liver and macrophages of the splenic red pulp, and is consistent with previous reports studying uptake of IONP in other systems [39, 62, 71, 72]. Interestingly, splenic iron accumulation exceeded hepatic levels in our study. This may reflect differences in macrophage recognition, splenic mechanical filtration mechanisms, and particle physicochemical properties. In particular, plasma protein adsorption following intravenous administration may increase the hydrodynamic size of the iron oxide nanoparticles, thereby enhancing splenic retention, as has been reported [73]. Future studies would aim to provide a more comprehensive understanding of organ-specific nanoparticle distribution. These investigations will include functional and molecular analyses of splenic immune cells, immunohistochemistry to visualize cellular localization of nanoparticles, and assessment of macrophage activation and function. Together, these approaches will clarify the mechanisms underlying preferential splenic accumulation, linking nanoparticle physicochemical properties, immune recognition, and tissue architecture, and will complement the current biodistribution findings.

To assess longer-term clearance, a separate group of rats received 12 mg Fe/kg sIONP, with organ collection conducted on days 14 and 28 (*n* = 3 per time point; vehicle controls were euthanized on day 28). As shown in Fig. 9b, liver iron levels remained significantly elevated on day 14 (0.545 ± 0.033 mg Fe/g dry tissue; *p* = 0.0031) but were no longer statistically different from controls by day 28 (0.399 ± 0.061 mg Fe/g dry tissue; *p* = 0.1239), This suggests progressive hepatic clearance. There was no statistically significant change in the kidney or heart iron level (Fig. 9b). Interestingly, spleen iron levels remained significantly elevated on day 14 (1.039 ± 0.071 mg Fe/g; *p* = 0.0230) and day 28 (1.092 ± 0.095 mg Fe/g; *p* = 0.0109), suggesting slower clearance or incorporation into splenic iron storage complexes. Chronic iron accumulation in the spleen has been associated with immune and hematologic effects including inflammatory responses, alterations in splenocyte function, oxidative stress, increase apoptosis, metabolic disturbances and immune modulations [74, 75]. Similarly, slow clearance of superparamagnetic iron oxide nanoparticles (AMI-25 or ferumoxtran-10) over periods of up to 7 weeks or 19 days has been reported [62, 76]. Notably, none of the animals exhibited behavioral or central nervous system effects, supporting a favorable safety profile for the sIONP tested. Extended-duration studies would further strengthen conclusions regarding long-term safety.

### Histopathology

Toxicity was assessed by histopathological evaluation of representative organs—liver, kidney, spleen, and heart — following systemic administration of sIONP. Animals received intravenous doses of 5, 12, or 20 mg Fe/kg and were assessed after 24 h, and additionally at 12 mg Fe/kg with assessment at 14 days and 28 days post-injection. At the time of assessment, animals were euthanized by CO₂ asphyxiation, and organs were harvested, fixed, and processed for hematoxylin and eosin (H&E) and Prussian blue (Perl’s) staining to evaluate tissue morphology and iron deposition. A certified pathologist assessed tissue sections for structural abnormalities and inflammation with comparisons to vehicle-treated controls.

**Fig. 10:**
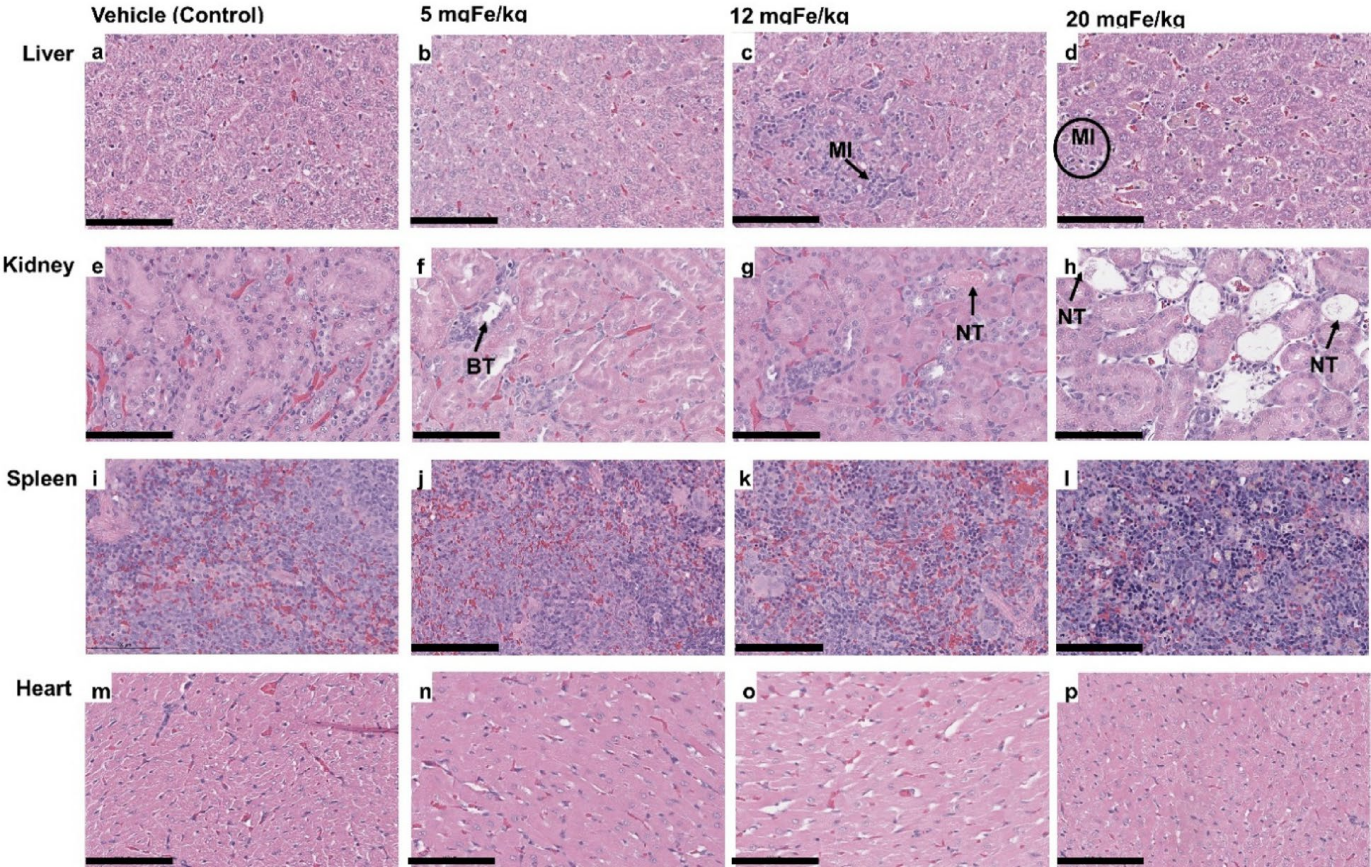
Histological sections of major organs at 24 h after systemic sIONP injection, stained with Hematoxylin and eosin (H&E). Representative images show sections of the liver: (**a**) vehicle control, (**b**) 5 mg Fe/kg, (**c**) 12 mg Fe/kg, and (**d**) 20 mg Fe/kg; kidney: (**e**) vehicle control, (**f**) 5 mg Fe/kg, (**g**) 12 mg Fe/kg, and (**h**) 20 mg Fe/kg; spleen: (**i**) vehicle control, (**j**) 5 mg Fe/kg, (**k**) 12 mg Fe/kg, and (**l**) 20 mg Fe/kg; heart: (**m**) vehicle control, (**n**) 5 mg Fe/kg, (**o**) 12 mg Fe/kg, and (**p**) 20 mg Fe/kg. Insets highlight pathological features, where “NT” indicates necrotic tubules, “BT” denotes basophilic tubules, and “MI” represents mononuclear infiltrates. Vehicle controls represent animals that did not receive sIONP. Scale bar: 100 μm for all panels

At 5 and 12 mg Fe/kg, hepatic architecture remained intact, with no evidence of necrosis, fibrosis, steatosis, or glycogen accumulation (Fig. 10b, c). However, mononuclear infiltrates were observed at 12 mg Fe/kg (Fig. 10c; Table S9). At 20 mg Fe/kg, liver sections showed sinusoidal congestion, focal portal vein dilation, rare megakaryocyte-like cells, erythroid micro clusters, and suspected extramedullary hematopoiesis (Fig. S5a, b). Scattered mononuclear infiltrates were also noted (Fig. 10d), suggestive of a dose-dependent immune response to sIONP exposure. Prussian blue staining revealed intracytoplasmic iron in Kupffer cells at 5, 12 and 20 mg Fe/kg (Fig. S3b, c, d) consistent with biodistribution result shown in Fig. 9a.

**Fig. 11:**
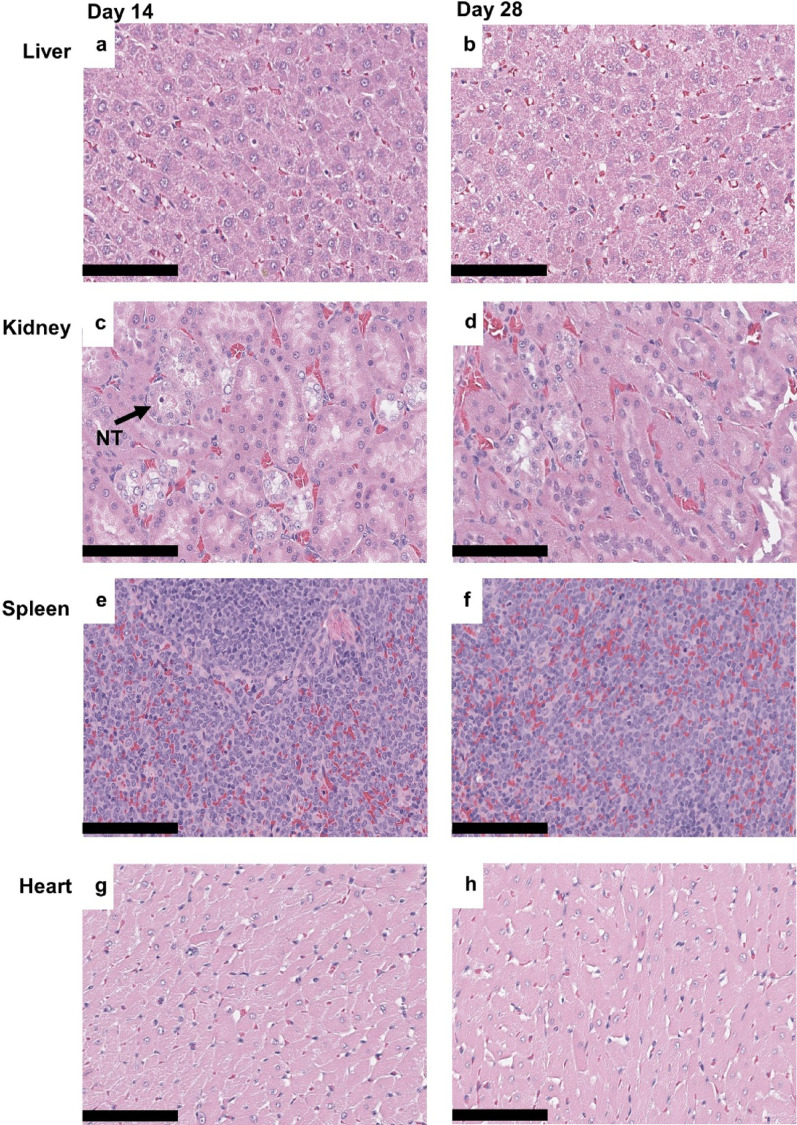
Hematoxylin and eosin (H&E)-stained histological sections of major organs at 14 and 28 days after injection of 12 mg Fe/kg sIONP. Representative images show sections of the liver: (**a**) day 14, (**b**) day 28; kidney: (**c**) day 14, (**d**) day 28; spleen: (**e**) day 14, (**f)** day 28; and heart: (**g**) day 14, (**h**) day 28. Inset labeled “NT” indicate necrotic tubule within the tissue. Scale bar:100 μm for all panels

In the 28-day study at 12 mg Fe/kg, mononuclear infiltrates in the liver were absent by day 14 and day 28 (Fig. 11a, b; Table S9), indicating resolution of immune activation and time-dependent clearance. Prussian blue staining confirmed progressive iron clearance from the liver (Fig. S4), consistent with the biodistribution results (Fig. 9b). Also, no fibrosis, steatosis, or glycogen accumulation was noted at later time points (Table S9).

At 24 h, kidneys from the 5 mg Fe/kg group exhibited minimal tubular basophilia without necrosis, inflammation, fibrosis, vacuolation, or glomerular abnormalities (Fig. 10f; Table S9). At 12 mg Fe/kg, minimal necrosis and basophilia were observed (Fig. 10g), while 20 mg Fe/kg induced moderate necrosis (Fig. 10h), epithelial loss in tubules, eosinophilic granular changes in the cytoplasm, minimal tubular vacuolation (Fig. S5c), and basophilia (Fig. S5d), without inflammation or glomerular abnormalities. These findings suggest dose-dependent, minimal-to-mild acute renal toxicity. Prussian blue staining revealed sparse iron deposits at 12 and 20 mg Fe/kg (Fig. S3g, h).

In the 28-day study at 12 mg Fe/kg group, minimal renal necrosis was present on day 14 (Fig. 11c) but resolved by day 28 (Fig. 11d). Minimal basophilia persisted, but no inflammation, fibrosis, vacuolation, or glomerular abnormalities were noted (Table S9).

No histopathological abnormalities were observed in the spleen (Fig. 10i–l) or heart (Fig. 10m–p) at any dose (5, 12, or 20 mg Fe/kg) or time point (24 h, day 14, or 28 at 12 mg Fe/kg; Fig. 11e–h). No necrosis, inflammation, hyperplasia, or fibrosis was detected. The myocardium appeared normal (Fig. 10m–p; Table S9). In the spleen, white pulp architecture was preserved, and hemosiderin-laden macrophages were noted in the red pulp (Fig. 10i–l). Prussian blue staining showed iron deposition in the spleen (Fig. S3i–l) but no detectable iron deposits in the heart (Fig. S3m–p), consistent with the biodistribution results in Fig. 9.

In conclusion, significant tissue morphological changes were observed, particularly in the liver and kidney at the highest dose (20 mg Fe/kg). The 28-day study at 12 mg Fe/kg demonstrated resolution of necrosis, mononuclear infiltrates, and as well as progressive clearance of iron, supporting that the observed acute effects are recoverable and reflect time-dependent sIONP clearance from major organs. Although the administered doses (5, 12, and 20 mg Fe/kg) far exceed the highest residual iron measured in cryopreserved organs (by six to seven orders of magnitude; Table 1), these findings provide important insights into sIONP biosafety and establish a robust foundation for further development of iron oxide nanoparticles for nanowarming-enabled cryopreservation in transplant medicine.

## Conclusion

Clinical application of nanowarming for organ preservation will require a thorough understanding of the biosafety of iron oxide nanoparticles (IONP). In this study, the in vivo toxicity profile of silica-coated iron oxide nanoparticles (sIONP) used in nanowarming was investigated. The physicochemical properties of sIONP were well-characterized, with a negative zeta potential of − 41 ± 3 mV, a hydrodynamic diameter of 86 ± 2 nm (DLS), and a size of 50 ± 2 nm (TEM).

Toxicity was assessed after intravenous injections at doses of 5, 12, and 20 mg Fe/kg, which are 6–7 orders of magnitude higher than equivalent dosing to the highest residual iron levels (3.36 ng Fe/kg) reported in the literature following organ cryopreservation (Table 1), and within the range of a theoretical worst-case (and unlikely to occur in a clinical setting) scenario in which a fully loaded kidney is transplanted (10.5–14 mg Fe/kg). Importantly, nanowarmed kidneys require unloading of both sIONP and cryoprotectants to be considered transplantable. A key strength of this study is the demonstration that tolerated administered doses can be 6–7 orders of magnitude higher than the estimated systemic exposure from residual sIONPs following organ transplantation (~ 3.36 ng Fe/kg). The use of systemic intravenous bolus administration was intentionally designed to model a theoretical worst-case exposure scenario in which the entire residual nanoparticle burden enters the circulation simultaneously. However, this approach does not fully recapitulate the clinical context of a transplanted organ containing residual nanoparticles. In this case nanoparticles may be partially retained within the organ vasculature, released gradually into circulation, interact with clotting factors, protein corona formation, biomolecular corona formation beyond proteins, endocrine system interactions, and organ-specific hemodynamics. Thus, systemic IV bolus administration likely represents a conservative overestimation of clinical systemic exposure. Further study of sIONP biodistribution after transplant of a vitrified and nanowarmed organ with residual nanoparticles remains to be done.

At 12 mg Fe/kg, sIONP exhibited an intermediate plasma half-life, volume of distribution, and clearance rate when compared to IONP used in MRI applications. Hematology and clinical chemistry revealed dose-dependent toxicity, with significant changes in blood parameters, kidney, and liver biomarkers at 20 mg Fe/kg, indicating anemia, immune response activation, renal impairment and liver injury 24 h post-injection. In contrast, at 12 mg Fe/kg, no significant increase or decrease in blood, liver, or kidney function markers were observed over 28 days, suggesting the safety of sIONP exposure at this dosing. All experimental and vehicle control animals showed similar steady weight gain and no abnormal behavior throughout the study period. Biodistribution analysis by ICP-MS and Prussian blue staining showed accumulation of sIONP in the liver and spleen, consistent with the expected systemic clearance. While the liver showed gradual clearance of sIONP deposits over 28 days, the spleen retained iron, suggesting that longer durations may be needed to achieve full clearance. H&E staining revealed morphological changes in the liver and kidney at 24 h after injection at 12 mg Fe/kg, which resolved by 28 days. These findings suggest that the residual sIONP levels typically observed after organ cryopreservation and transplantation (Table 1) could pose no overt toxicity. However, findings at 20 mg Fe/kg warrant further study of the immune response. Additionally, persistent iron in the spleen suggests the necessity of long-term studies beyond 28 days.

This study provides an application-specific in vivo safety evaluation of sIONP in the context of organ nanowarming. The dose-dependent toxicity observed here demonstrates tolerance at ≤ 12 mg Fe/kg and acute toxicity at 20 mg Fe/kg is broadly consistent with prior reports of iron oxide nanoparticles [69, 75, 77]. Thus, the primary contribution of this work lies not in identifying novel toxicological mechanisms, but in establishing a quantitative safety margin directly relevant to the nanowarming technique. Specifically, these data define systemic dose thresholds relative to the estimated residual nanoparticle exposure following organ transplantation, thereby providing translational safety validation for clinical implementation. While systemic administration, as performed in this study, provides valuable insight into the isolated effects of sIONP toxicity, there are some limitations of this work. With respect to sample size, we recognize that the number of animals used was limited. Nevertheless, the observed trends were consistent and clear, thereby achieving the goals of this application specific safety study. Future studies with expanded animal groups can confirm and extend these findings to answer mechanistic questions. For instance, further work could directly evaluate the impact of sIONP on transplanted organs, investigate the immune pathways potentially activated by sIONP, and assess toxicity over longer timeframes. Although this study did not determine the lowest detectable lethal dose (LDL), this was considered unnecessary since our findings demonstrate safety at exposure levels orders of magnitude higher than the anticipated clinical exposure to residual iron following nanowarming and cryopreservation. Furthermore, comprehensive toxicity profiling of IONPs with diverse surface chemistry, shapes, sizes, and coatings will be essential to ensure the safe clinical translation of nanowarming technologies.

## Experimental section

### Materials

Ferrofluid EMG308 (Ferrotec Inc., Livermore, CA), polyvinylpyrrolidone (PVP-10, MW 10 kDa, Thermo Scientific, Waltham, MA), 2-[methoxy(polyethyleneoxy)-propyl]9-12-trimethoxysilane (PEG-silane, Gelest Inc., Morrisville, PA), ammonium hydroxide (NH₄OH, 28%, VWR Inc., Radnor, PA), ethanol (95% and 99%, Decon Labs, King of Prussia, PA), tetraethyl orthosilicate (TEOS, Sigma-Aldrich, Burlington, MA), chlorotrimethylsilane (TMS, > 99%, Sigma-Aldrich, Burlington, MA), and 0.9% normal saline Injection, USP (ICU Medicals, San Clemente, CA) were all used as received. Male Sprague-Dawley rats were obtained from Charles River Laboratories (Wilmington, MA).

### sIONP synthesis

Silica-coated iron oxide nanoparticles (sIONP) were synthesized following an established protocol [12]. Briefly, 48 g of PVP-10 was completely dissolved in 407 mL of ultrapure water at 23 ± 2 °C. A ferrofluid solution (EMG308, Ferrotec) containing 1.44 g of iron was added to the PVP solution, yielding a final volume of 431 mL. This mixture was probe-sonicated (Q500, Qsonica) at 35% amplitude, in 4-second on / 2-second off cycles for 45 min under continuous stirring. The resulting dispersion was transferred to a 4 L vessel containing 3.2 L of 95% ethanol and sonicated again under identical conditions. The reaction mixture was then moved to a 4 L glass reaction vessel (Wilmad-LabGlass, LG-8082-104) and stirred continuously using an overhead mechanical stirrer (OS 20-S, Waverly). Subsequently, 160 mL of ammonium hydroxide was added, followed by sequential additions of TEOS (80 mL), PEG-silane (15 mL after 1 h), and finally, TMS (2.25 mL) after another 30 min. The reaction proceeded for 24 h under constant stirring. The final product was concentrated under reduced pressure and collected for purification.

### sIONP purification

Purification was performed using a previously reported method involving a tangential flow filtration (TFF) system (ÄKTA™ flux, GE Healthcare Bioscience Corp, USA) with Xampler ultrafiltration cartridges (UFP-100 C-4 × 2MA, Cytiva, Marlborough, MA) [13]. The synthesized sIONP (1.44 gFe) was purified using the TFF parameters in our previous report [13]. sIONP solution was transferred into the feed at a flow rate of 120 mL/min, underwent diafiltration, during which the retentate was circulated through the cartridge and the feed reservoir at the flow rate of 300 ml/min with a continuous supply of ultra-pure water (12 L) from the external reservoir at a flow rate of 120 ml/min, with constant volume maintenance at 400 mL. At the end, the system was set into concentration mode, during which the sIONP was concentrated, collected, characterized, and stored for in vivo administration.

### Characterization of sIONP

Zeta potential and dynamic light scattering (DLS) measurements were conducted using a ZetaPALS instrument (Brookhaven Instruments Corp.) equipped with a 635 nm diode laser. Transmission electron microscopy (TEM) was performed using a Tecnai T12 microscope (FEI, USA) operating at 120 kV, with samples prepared on 200-mesh carbon-coated copper grids. Prior to in vivo use, purified sIONP were filtered through a 1 μm pluriStrainer (pluriSelect, 43-50001-01), quantified by inductively coupled plasma optical emission spectrometry (ICP-OES, iCAP 6500, Thermo Fisher Scientific) using a 1150 W power setting to achieve final dosing concentrations of 5, 12, or 20 mg Fe/kg, and sterilized via X-ray irradiation (X-Rad 320, Precision X-Ray at 320 kV, 12.5 mA, and a dose of 10,000 centigray) to ensure sterility for intravenous administration.

### Animal care and ethics

All animal procedures were conducted in accordance with institutional guidelines maintaining relevant ethical considerations and were approved by the Institutional Animal Care and Use Committee (IACUC) of the University of Minnesota (Protocol #2204–39970 A). Rats (6–8 weeks old) with pre-installed exteriorized catheters in the jugular and femoral veins were procured from Charles River Laboratories (USA). Rats were acclimatized for a minimum of 72 h before the start of the study. They were housed in a facility with a 12 h light / 12 h dark cycle, maintained at an ambient temperature of 64–72 °F (18–22 °C) and relative humidity of 30–50%, with ad libitum access to food and water.

### Animal injection and monitoring

Notably, vehicle control animals underwent identical anesthesia, catheterization, monitoring, and handling procedures as sIONP-treated groups. Control animals received the corresponding vehicle solution without sIONPs to ensure procedural consistency across groups. Before dosing, each rat was weighed and its body weight recorded. Rats received an intravenous (IV) injection of sIONP at 5, 12 or 20 mg Fe/kg colloidally suspended in 0.9% sodium chloride solution (2 µL/g body weight). Vehicle controls received equivalent volumes of sterile 0.9% sodium chloride injection only. Following injection, all animals were monitored daily for food intake, behavior, body-weight changes, and overall health to assess any signs of toxicity or adverse effects.

### Plasma pharmacokinetics

At 12 mg Fe/kg, sIONP in 0.9% sodium chloride injection (2 µL/g body weight) were intravenously injected into the rats. Under brief anesthesia, 300 µL blood samples were drawn from the jugular catheter at 5, 30, 60, and 120 min post-injection into heparinized tubes. Plasma was isolated immediately by centrifugation, processed, and iron concentrations were measured by Inductively Coupled Plasma Mass Spectrometry (ICP-MS). Notably, rats were anesthetized only for the duration of blood sample collections. Pharmacokinetic parameters, including volume of distribution (Vd_sIONP_), plasma clearance (CL _sIONP_), and half-life (t_1/2_), were determined individually for each rat.

### Hematology and clinical chemistry

sIONP in 0.9% normal saline were intravenously injected (2 µL/g body weight) into rats at a 5, 12, and 20 mg Fe/kg. Vehicle controls received 0.9% normal saline injection only. At 24 h post-injection, 500 µL of blood was drawn for hematology and 750 µL for clinical chemistry (biochemistry, electrolytes, and glucose). Furthermore, time-dependent evaluation of hematology and clinical chemistry was determined over a 28-day period at a 12 mg Fe/kg dose and vehicle control. Blood (500 µL for hematology and 750 µL for chemistry) was collected on days 7, 14, 21, and 28 after injection. On day 14, three sIONP-treated rats were euthanized for organ iron quantification and histopathology while the remaining three and all vehicle controls continued through day 28. Hematology and clinical chemistry assays were performed by the Clinical Pathology Laboratory, College of Veterinary Medicine, University of Minnesota.

### Biodistribution

To assess sIONP biodistribution, rats received intravenous injections (2 µL/g body weight) of sIONP in 0.9% normal saline injection at 5, 12, or 20 mg Fe/kg. Vehicle controls received normal saline injections only. Twenty-four hours post-injection, all rats were euthanized by CO₂ asphyxiation. Liver, kidney, spleen, and heart were recovered, weighed, dried completely in a vacuum oven at 120 °C, and then powdered. Iron concentration in the organs was determined using ICP-MS at ALS Global with an Agilent 7700 ICP-MS instrument. Each organ sample was placed into a Teflon digestion vessel with 15% concentrated nitric acid and 5% concentrated hydrochloric acid. The digestion vessels were sealed and placed in a 105 °C oven for a minimum of 12 h. After cooling, the samples were transferred to centrifuge tubes and diluted to a final volume of 20 mL with deionized water. Iron concentration was then measured by ICP-MS, and the results were recorded.

Biodistribution and clearance of sIONP (12 mg Fe/kg) from the organs over a 28-day period post-injection was further evaluated. Three sIONP-treated rats were euthanized on day 14 and three on day 28 (all by CO₂ asphyxiation), with vehicle controls euthanized on day 28. Organs were processed and analyzed identically to the 24-hour post-injection study.

### Histopathology

To observe pathological changes and iron deposition in the organs, sections of the liver, kidney, spleen, and heart were recovered from euthanized rats at 24 h (5, 12, and 20 mg Fe/kg) and 28 day-period (days 14 and 28, 12 mg Fe/kg) and compared to vehicle control. All organs were fixed in 10% neutral-buffered formalin, processed, paraffin-embedded, sectioned, and mounted on glass slides. Sections were stained with hematoxylin and eosin (H&E) to evaluate general histopathology, and Perls’ Prussian blue to visualize iron deposition. Slides were digitally scanned (GT 450, Leica Biosystems, Inc.) to generate whole-slide images, which were reviewed by a certified pathologist at HistoWiz, USA.

### Statistical analysis

Statistical analyses were performed using GraphPad Prism (version 10.2.0). Normality of continuous variables was assessed using the Shapiro–Wilk test, quantile–quantile (Q–Q) plots, and frequency distribution histograms. Data are presented as mean ± standard deviation (SD). The number of replicates and specific statistical tests used are indicated in the corresponding figure legends. Homogeneity of variance was evaluated using the F-test or Brown–Forsythe test. For comparisons between normally distributed groups with equal variances, a two-tailed unpaired *t*-test was used for two-group comparisons. Comparisons across multiple time points were performed using multiple *t*-tests, with *p*-values adjusted for multiple comparisons using the Holm–Šidák method. Dose-dependent effects were analyzed using one-way ANOVA followed by Tukey’s honestly significant difference (HSD) post hoc test for multiple comparisons. For non-normally distributed variables, comparisons were performed using the Mann–Whitney U test. All statistical tests were two-sided, and a *p*-value < 0.05 was considered statistically significant. *p*-values were adjusted to account for multiple comparisons.

## Supplementary material

Supplementary material 1.

## Data Availability

The datasets supporting the conclusions of this manuscript are included within the article and its supplementary file.
