# Supplementary material for: In vivo toxicity assessment of silica-coated iron oxide nanoparticles for nanowarming in organ cryopreservation
Source: J Nanobiotechnology. 2026 May 7;24:603. doi: 10.1186/s12951-026-04471-6 (PMC13330078; doi:10.1186/s12951-026-04471-6)
Supplement: Supplementary file 1 — Supplementary material 1. [file 12951_2026_4471_MOESM1_ESM.docx]

**Supplementary Information**

***In Vivo* Toxicity Assessment of Silica-Coated Iron Oxide Nanoparticles for Nanowarming in Organ Cryopreservation**

Onyinyechukwu Justina Oziri^1^, Cameron Scheithauer^1^, Henry L Wong^2^, Michael L Etheridge^1^, Erik B Finger^3^*, John C Bischof^1,4,5^*

^1^ Department of Mechanical Engineering, University of Minnesota, Minneapolis, MN, USA

^2^Institute for Therapeutics Discovery and Development, University of Minnesota, MN, USA

^3^ Department of Surgery, University of Minnesota, Minneapolis, MN, USA

^4^ Department of Biomedical Engineering, University of Minnesota, Minneapolis, MN, USA

^5^ Institute for Engineering in Medicine, University of Minnesota, Minneapolis, MN, USA

* Corresponding authors: [bischof@umn.edu](mailto:bischof@umn.edu) and [efinger@umn.edu](mailto:efinger@umn.edu)


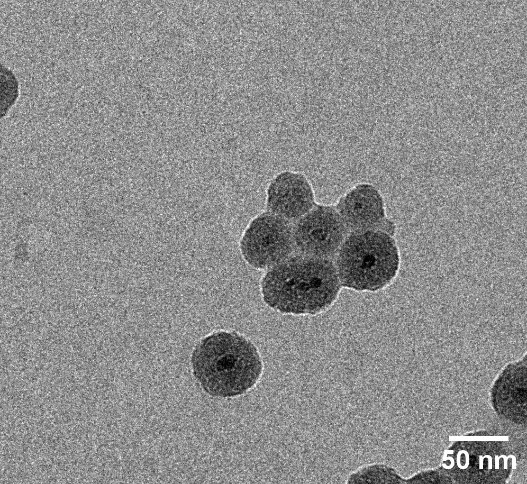


86 ± 2 nm

(a)

(b)

**Figure S1. Physicochemical characterization of sIONP.** (a) Hydrodynamic diameter (86 ± 2 nm) and zeta potential (−41 ± 3 mV) measured by dynamic light scattering (DLS). (b) Size determined by transmission electron microscopy (TEM; 50 ± 2 nm). Blue indicates the silica, PVP, and PEG coatings surrounding the iron oxide nanoparticle core.

**Table S1.**  **Summary of residual iron levels and estimations across studies and organs.**

| Organ | Nanoparticle | Animal body weight (g) | Estimated organ wet weight (g) | Estimated organ dry weight (g) | Residual iron in the organ (µg Fe / dry weight) | Estimated residual iron in the organ (µg Fe /wet weight) | Estimated systemic dosing (ng Fe/kg) | References |
| --- | --- | --- | --- | --- | --- | --- | --- | --- |
| Kidney | sIONP | 200-250 | 1.75 | 0.35 | 2.0 | 0.40 | 2.80 | [1] |
| Kidney | sIONP | 176-300 | 2.10 | 0.42 | 2.4 | 0.48 | 3.36 | [2] |
| Kidney | sIONP | 450-525 | 3.68 | 0.74 | 1.23 | 0.25 | 1.75 | [3] |
| Heart | sIONP | 250-300 | 1.26 | 0.30 | 1.2 | 0.24 | 1.20 | [4] |
| *Heart | SPION | 300 | - |  | 10.2 |  | 31.0 | [5] |

* Published values extracted from [5].

**Estimated organ wet weight (g):**

- Kidney = 0.7% of animal body weight [6]
- Heart = 0.42 % of animal body weight [7]

*(Note: The highest animal body weight was used for these estimations.)*

**Estimated organ dry weight (g):**

- Assuming water content:
  - Kidney: 80% of wet weight
  - Heart: 76.1% of wet weight
- Calculation:

$$Organ dry weight =Organ wet weight -Water fraction of the organ$$

Estimated residual iron in the organ (mg Fe / wet weight):

$$Residual Fe \left( mg Fe/wet weight \right)= \frac{Residual Fe (mg Fe/dry weight (g)) \times Estimated organ dry weight (g)}{Estimated organ wet weight (g)}$$

Estimated systemic dosing (mg Fe / kg body weight):

$$Estimated systemic dosing (mg Fe/kg body weight) =mg Fe/ organ wet weight \times\frac{organ wet weight}{animal body weight}$$

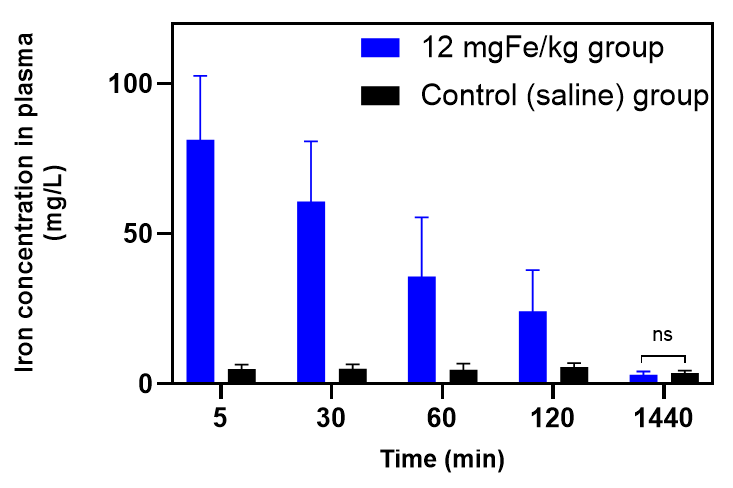

**Figure S2. Plasma iron concentration over time following 12 mg Fe/kg** **sIONP intravenous injection in rats.** Plasma iron levels (mg/mL) measured over time after intravenous injection of sIONP (12 mg Fe/kg) or vehicle control in male Sprague-Dawley rats (n = 3).

**Table S2.** **Statistical summary of hematology and biochemical indicators of kidney and liver functions at 24 h post-injection of 5, 12, and 20 mg Fe/kg** **sIONP.**

| **Hematology** | **P-Values**  *sIONP Dose* | | |
| --- | --- | --- | --- |
|  | *5 mg Fe/kg* | *12 mg Fe/kg* | *20 mg Fe/kg* |
| White blood cells | 0.8885 | 0.5845 | 0.9983 |
| Red blood cells | 0.8792 | 0.7109 | 0.0514 |
| Hemoglobin | 0.4319 | 0.9116 | 0.0030** |
| Mean corpuscular volume | 0.9590 | 0.8371 | 0.9978 |
| Mean corpuscular hemoglobin | 0.9782 | 0.7528 | 0.9706 |
| Mean corpuscular hemoglobin concentration | > 0.9999 | 0.8666 | 0.6848 |
| Platelets | 0.8978 | 0.9322 | 0.8757 |
| Hematocrit | 0.5090 | 0.8028 | 0.0049** |
| Percentage neutrophils | 0.6595 | 0.9966 | 0.0001*** |
| Absolute neutrophil count | 0.7461 | 0.7981 | 0.0034** |
| Percentage lymphocytes | 0.5965 | 0.9999 | 0.0005*** |
| Absolute lymphocyte count | 0.9840 | 0.4906 | 0.0831 |
| **Biochemical markers** | | | |
| BUN | 0.9394 | 0.8559 | ˂0.0001**** |
| Creatinine | > 0.9999 | 0.9882 | ˂0.0001**** |
| Total protein | 0.9015 | 0.0297* | 0.5535 |
| Albumin | 0.5034 | 0.0755 | 0.5034 |
| Alkaline Phosphatase | 0.9990 | 0.5584 | 0.9794 |
| Alanine Aminotransferase | 0.9992 | > 0.9999 | 0.0020** |
| Aspartate Aminotransferase | 0.9594 | 0.3054 | 0.9843 |
| Total bilirubin | 0.5252 | > 0.9999 | > 0.9999 |

*p*-values comparing vehicle control (n = 3) to sIONP treatment groups (5, 12, and 20 mg Fe/kg; n = 9 total, 3 per dose) for hematological (Figure 3) and biochemical parameters (Figure 4) measured 24 hours post-injection analyzed using one-way ANOVA followed by Tukey’s post hoc test. *p ˂ 0.05, **p ˂ 0.01, ***p ˂0.001, and ****p ˂0.0001.

**Table S3. Statistical summary of electrolyte and glucose levels at 24 h post-injection of 5, 12, and 20 mg Fe/kg; sIONP.**

| **Electrolytes and glucose** | **P-Values**  *Dose* | | |
| --- | --- | --- | --- |
|  | *5 mg Fe/kg* | *12 mg Fe/kg* | *20 mg Fe/kg* |
| Sodium | 0.9910 | 0.5097 | 0.0026** |
| Chloride | 0.8795 | 0.1996 | 0.0009*** |
| Phosphorus | 0.9021 | 0.2639 | 0.0004*** |
| Bicarbonate | 0.7570 | 0.8877 | >0.9999 |
| Potassium | 0.1896 | 0.8652 | 0.0002*** |
| Calcium | 0.9764 | 0.9764 | 0.0987 |
| Glucose | 0.9962 | 0.7958 | 0.9446 |

*p*-values comparing vehicle control (n = 3) to sIONP treatment groups (5, 12, and 20 mg Fe/kg; n = 3 per dose) for electrolyte and glucose measurements (Figure 5) 24 hours post-injection analyzed using one-way ANOVA followed by Tukey’s post hoc test. *p ˂ 0.05, **p ˂ 0.01, and ***p ˂0.001.

**Table S4. Statistical summary of hematology following 12 mg Fe/kg** **sIONP administration over 28 days.**

| **Hematology** | **P-Values**  *Days* | | | |
| --- | --- | --- | --- | --- |
|  | *7* | *14* | *21* | *28* |
| White blood cells | 0.9838 | 0.3314 | 0.9945 | 0.9312 |
| Red blood cells | 0.6270 | 0.8645 | 0.3593 | 0.0464* |
| Hemoglobin | 0.3967 | 0.6004 | 0.3967 | 0.0270* |
| Mean corpuscular volume | 0.7291 | 0.8806 | 0.8806 | 0.7291 |
| Mean corpuscular hemoglobin | 0.5244 | 0.5244 | 0.8761 | 0.6348 |
| Mean corpuscular hemoglobin concentration | 0.5144 | 0.2626 | 0.2625 | 0.0564 |
| Platelets | 0.5239 | 0.6541 | 0.9821 | 0.6541 |
| Hematocrit | 0.3646 | 0.9813 | 0.3646 | 0.0727 |
| Percentage neutrophils | 0.9406 | 0.0866 | 0.9406 | 0.6983 |
| Absolute neutrophils count | 0.8836 | 0.0310* | 0.8836 | 0.7368 |
| Percentage lymphocyte | 0.9246 | 0.0120* | 0.9246 | 0.9246 |
| Absolute lymphocyte count. | 0.9925 | 0.8417 | 0.9925 | 0.9925 |

*p*-values comparing vehicle control (n = 3) with sIONP-treated rats (12 mg Fe/kg; n = 6 on days 7 and 14, n = 3 on days 21 and 28) for hematological parameters (Figure 6) measured at each time point analyzed and using multiple *t*-tests, with *p*-values adjusted for multiple comparisons using the Holm–Šidák method. *p ˂ 0.05.

**Table S5. Statistical summary of biochemical indicators of kidney and liver function following 12 mg Fe/kg** **sIONP administration over 28 days.**

| **Biochemical markers** | **P-Values**  *Days* | | | |
| --- | --- | --- | --- | --- |
|  | *7* | *14* | *21* | *28* |
| BUN | 0.0297* | 0.9028 | 0.9028 | 0.0145* |
| Creatinine | 0.8463 | 0.5177 | > 0.9999 | > 0.9999 |
| Total protein | 0.9212 | 0.9283 | 0.1047 | 0.4280 |
| Albumin | 0.9546 | > 0.9999 | 0.2561 | 0.2561 |
| Alkaline Phosphatase | 0.7646 | 0.7646 | 0.7646 | 0.9655 |
| Alanine Aminotransferase | 0.7731 | 0.7731 | 0.3005 | 0.2078 |
| Total bilirubin | > 0.9999 | 0.8025 | > 0.9999 | > 0.9999 |
| Aspartate Aminotransferase | 0.0744 | 0.6240 | 0.6240 | 0.4653 |

*p*-values comparing vehicle control (n = 3) with sIONP-treated rats (12 mg Fe/kg; n = 6 on days 7 and 14, n = 3 on days 21 and 28) for serum biochemical parameters (Figure 7) measured at each time point and analyzed multiple *t*-tests, (a, c, d, e, f, h) or Mann–Whitney *U* (b, g) with *p*-values adjusted for multiple comparisons using the Holm–Šidák method. *p ˂ 0.05.

**Table S6. Statistical summary of electrolyte and glucose levels following 12 mg Fe/kg** **sIONP administration over 28 days.**

|  | **P-Values**  *Days* | | | |
| --- | --- | --- | --- | --- |
|  | *7* | *14* | *21* | *28* |
| Sodium | 0.3767 | 0.5248 | 0.1350 | 0.4744 |
| Chloride | 0.7359 | 0.6290 | 0.7359 | 0.7359 |
| Phosphorus | 0.3485 | 0.2713 | 0.4716 | 0.0061** |
| Bicarbonate | 0.6795 | 0.6795 | 0.6411 | 0.6412 |
| Potassium | 0.5855 | 0.5160 | 0.2671 | 0.0114* |
| Calcium | 0.3465 | 0.3465 | 0.3465 | 0.0249* |
| Glucose | 0.9306 | 0.9306 | 0.9306 | 0.9306 |

*p*-values comparing vehicle control (n = 3) with sIONP-treated rats (12 mg Fe/kg; n = 6 on days 7 and 14, n = 3 on days 21 and 28) for electrolyte and glucose measurements (Figure 8) at each time point and analyzed using multiple *t*-tests, with *p*-values adjusted for multiple comparisons using the Holm–Šidák method. *p ˂ 0.05 and **p ˂ 0.01.

**Table S7. Statistical summary of sIONP biodistribution at 24 h post-injection of 5, 12, and 20 mg Fe/kg (Figure 9a).**

| **Doses**  **(mg Fe/kg)** | **P-Values**  *Organs* | | | |
| --- | --- | --- | --- | --- |
|  | *Liver* | *Kidney* | *Spleen* | *Heart* |
| 5 | 0.1772 | 0.3475 | 0.6718 | 0.1105 |
| 12 | 0.0034** | 0.6770 | 0.5009 | 0.6129 |
| 20 | ˂ 0.0001**** | 0.9728 | 0.0003*** | 0.0431* |

*p*-values comparing vehicle control (n = 3) with sIONP-treated groups (5, 12, and 20 mg Fe/kg; n = 3 per dose) for iron accumulation in organs measured 24 hours post-injection, analyzed using one-way ANOVA followed by Tukey’s post hoc test. *p ˂ 0.05, **p ˂ 0.01, ***p ˂0.001, and ****p ˂0.0001.

**Table S8. Statistical summary of sIONP biodistribution following 12 mg Fe/kg administration (Figure 9b).**

| **Time after injection**  **(Days)** | **P-Values**  *Organs* | | | |
| --- | --- | --- | --- | --- |
|  | *Liver* | *Kidney* | *Spleen* | *Heart* |
| 14 | 0.0031** | 0.3368 | 0.0230* | 0.1109 |
| 28 | 0.1239 | 0.4898 | 0.0109* | 0.9976 |

*p*-values comparing vehicle control (n = 3) with sIONP-treated rats (12 mg Fe/kg; n = 6 total, 3 per time point) for iron accumulation in organs measured over 28 days, analyzed using a two-tailed unpaired t-test. *p ˂ 0.05 and **p ˂ 0.01

**Table S9:** Pathological analysis and score according based on the National Toxicology Program.

| 1. **LIVER** | | | | | | |  |
| --- | --- | --- | --- | --- | --- | --- | --- |
| **Dose (mgFe/kg)** | **Time** | **Necrosis** | **Mononuclear**  **Infiltrates** | **Fibrosis** | **Steatosis** | **Glycogen**  **accumulation** |  |
| 0 (control) |  | 0 | 0 | 0 | 0 | 0 |  |
| 5 | 24 h. | 0 | 0 | 0 | 0 | 0 |  |
| 12 | 24 h. | 0 | 1 | 0 | 0 | 0 |  |
| 20 | 24 h. | 0 | 1 | 0 | 0 | 0 |  |
|  | | | | | | |  |
| 12 | Day 14 | 0 | 0 | 0 | 0 | 0 |  |
| 12 | Day 28 | 0 | 0 | 0 | 0 | 0 |  |
| 1. **KIDNEY** | | | | | | |  |
| **Dose (mg Fe/kg)** |  | **Necrosis** | **Inflammation** | **Fibrosis** | **Tubular**  **Basophilia** | **Tubular**  **Vacuolation** | **Glomerular**  **Abnormalities** |
| 0 (control) |  | 0 | 0 | 0 | 0 | 0 | 0 |
| 5 | 24 h. | 0 | 0 | 0 | 1 | 0 | 0 |
| 12 | 24 h. | 1 | 0 | 0 | 1 | 0 | 0 |
| 20 | 24 h. | 3 | 0 | 0 | 1 | 1 | 0 |
|  | | | | | | | |
| 12 | Day 14 | 1 | 0 | 0 | 1 | 0 | 0 |
| 12 | Day 28 | 0 | 0 | 0 | 1 | 0 | 0 |
| 1. **SPLEEN** | | | | | |  | |
| **Dose (mg Fe/kg)** |  | **Necrosis** | **Inflammation** | **Fibrosis** | **Hyperplasia** |  |  |
| 0 (control) |  | 0 | 0 | 0 | 0 |  |  |
| 5 | 24 h. | 0 | 0 | 0 | 0 |  |  |
| 12 | 24 h. | 0 | 0 | 0 | 0 |  |  |
| 20 | 24 h. | 0 | 0 | 0 | 0 |  |  |
|  | | | | | |  |  |
| 12 | Day 14 | 0 | 0 | 0 | 0 |  |  |
| 12 | Day 28 | 0 | 0 | 0 | 0 |  |  |
| 1. **HEART** | | |  | |  |  |  |
| **Dose (mg Fe/kg)** |  | **Necrosis** | **Inflammation** | **Hyperplasia** |  |  |  |
| 0 (control) |  | 0 | 0 | 0 |  |  |  |
| 5 | 24 h. | 0 | 0 | 0 |  |  |  |
| 12 | 24 h. | 0 | 0 | 0 |  |  |  |
| 20 | 24 h. | 0 | 0 | 0 |  |  | |
|  | | | | |  |  |  |
| 12 | Day 14 | 0 | 0 | 0 |  |  |  |
| 12 | Day 28 | 0 | 0 | 0 |  |  |  |

**Grade and severity**: 0- normal, 1- minimal, 2- Mild, 3- moderate, 4-marked, 5-severe

**Portion of affected tissue** (%): 0: 0, 1: 1-10, 2: 11-25, 3: 26-50, 4: 51-75, 5: >76

**Quantifiable finding (Foci):** 0: 0, 1: 1-2, 2: 3-6, 3: 7-12, 4: >12, 5: Diffuse


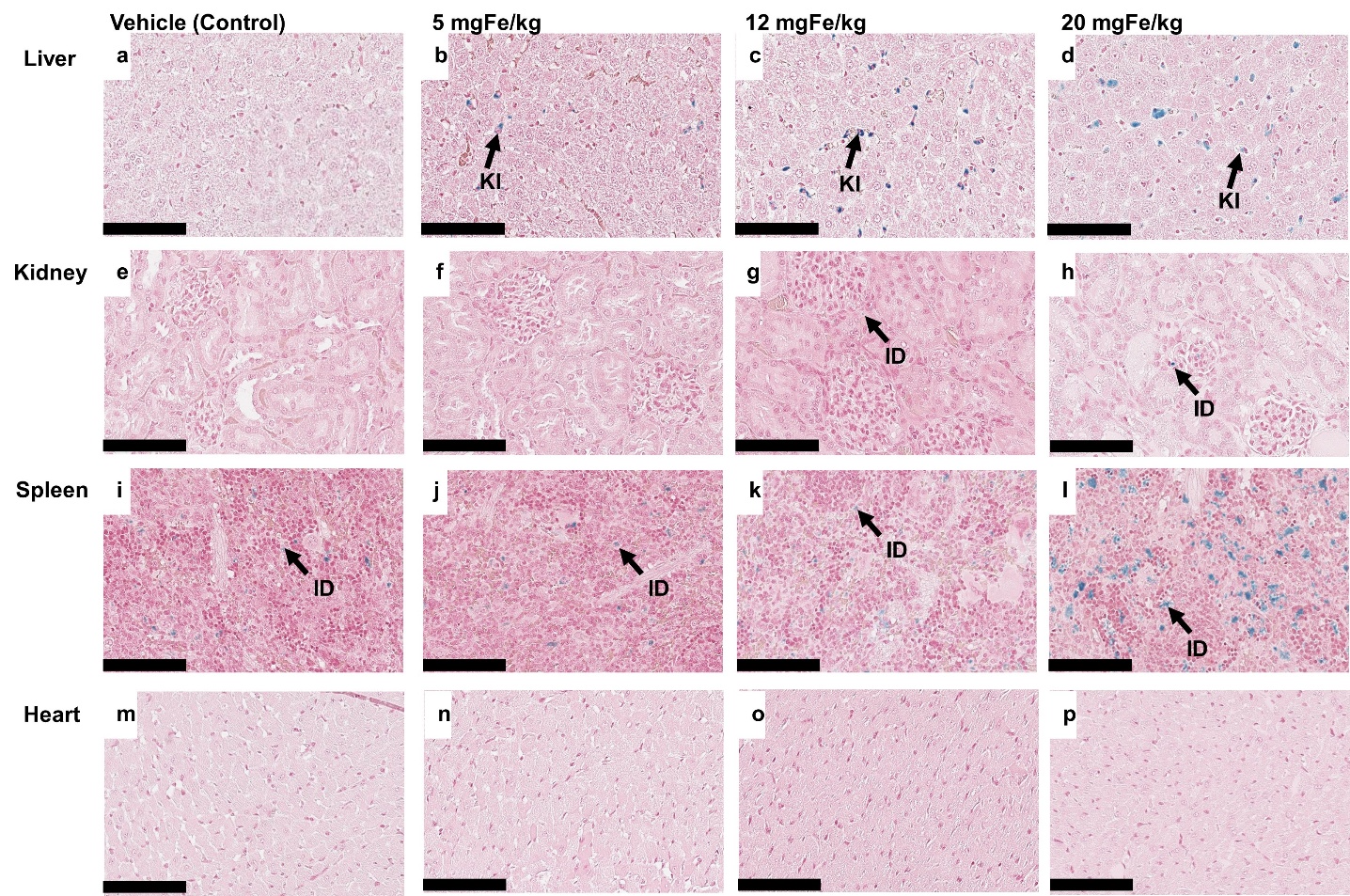


**Figure S3. Histological sections of major organs at 24 hours after systemic sIONP injection, stained with Prussian blue*.*** Representative images show sections of the liver: (a) vehicle control, (b) 5 mg Fe/kg, (c) 12 mg Fe/kg, and (d) 20 mg Fe/kg; kidney: (e) vehicle control, (f) 5 mg Fe/kg, (g) 12 mg Fe/kg, and (h) 20 mg Fe/kg; spleen: (i) vehicle control, (j) 5 mg Fe/kg, (k) 12 mg Fe/kg, and (l) 20 mg Fe/kg; heart: (m) vehicle control, (n) 5 mg Fe/kg, (o) 12 mg Fe/kg, and (p) 20 mg Fe/kg. Iron deposits appear as blue staining. Insets labeled “KI” indicate iron-containing Kupffer cells, and “ID” denotes iron deposition within the tissue. Vehicle controls represent animals that did not receive sIONP. Scale bar: 100 μm for all panels.

**
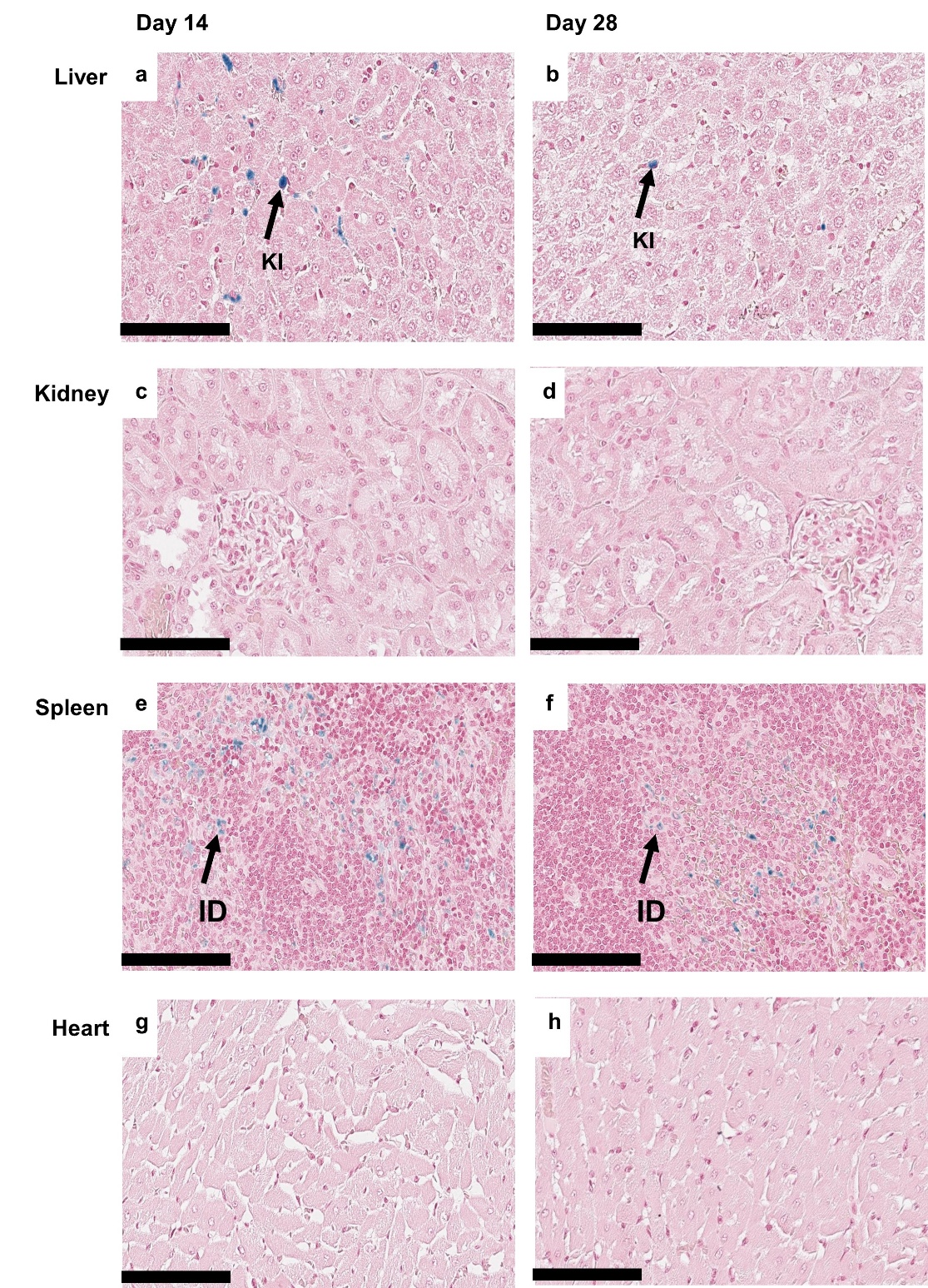
**

**Figure S4. Prussian, blue-stained histological sections of major organs at 14 and 28 days after injection of 12 mg Fe/kg sIONP.** Representative images show sections of the liver: (a) day 14, (b) day 28; kidney: (c) day 14, (d) day 28; spleen: (e) day 14, (f) day 28; and heart: (g) day 14, (h) day 28. Iron deposits appear as blue staining. Insets labeled “KI” indicate iron-containing Kupffer cells, and “ID” denotes iron deposition within the tissue. Scale bar:100 μm for all panels.


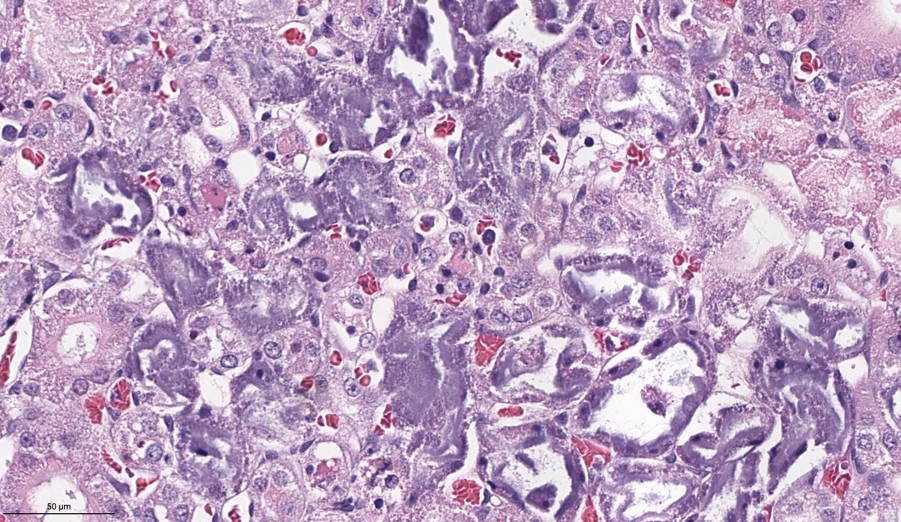


**NT**

**BT**

**BT**


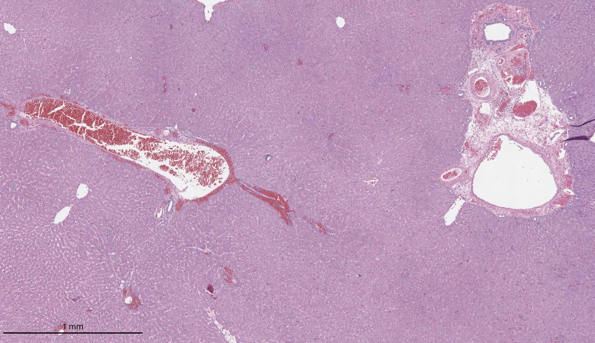


**SC**

**DPV**


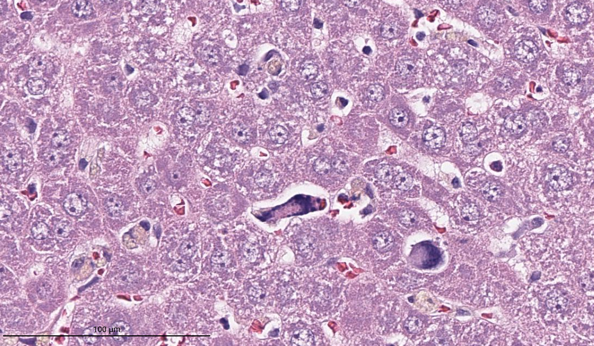


**RMC**


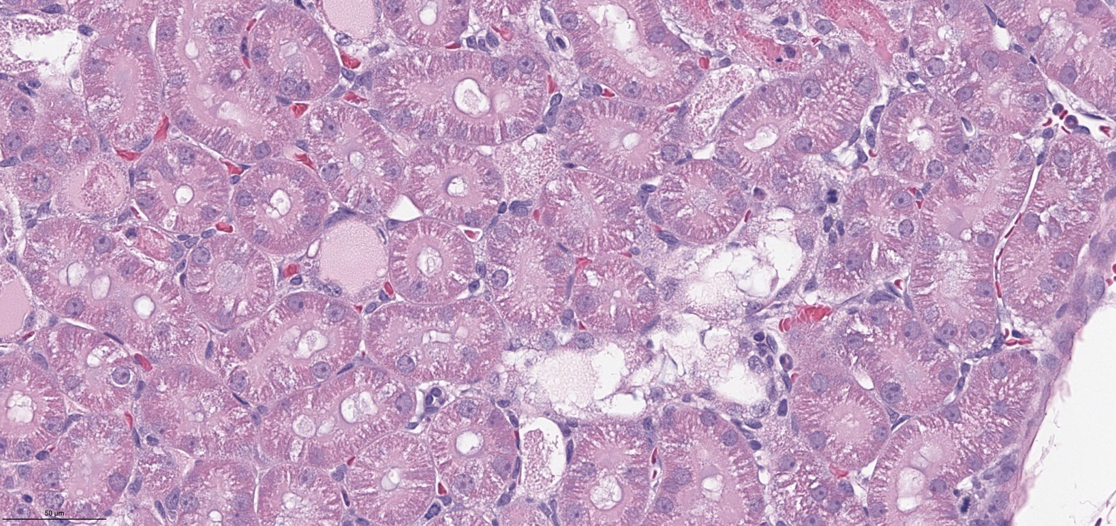


**TV**

**Liver**

**Kidney**

**a**

**b**

**c**

**d**

**Figure S5.** **Hematoxylin and eosin (H&E)-stained histological sections of major organs at 24 hours after systemic sIONP injection at 20 mg Fe/kg.** Representative images show sections of the liver with: (a) sinusoidal congestion (SC), and (b) atypical cells with hyperchromatic nuclei, resembling megakaryocyte-like cells (RM). Kidney sections show: (c) tubular vacuolation (TV), and (d) basophilic tubules (TB) and necrotic tubules (NT). Scale bars: (a) 1 μm, (b) 100 μm, (c) 50 μm, and (d) 50 μm.

**References**

[1] Z. Gao *et al.*, "Preparation of Scalable Silica-Coated Iron Oxide Nanoparticles for Nanowarming," *Advanced Science,* vol. 7, no. 4, Feb 2020, Art no. 1901624, doi: 10.1002/advs.201901624.

[2] A. Sharma *et al.*, "Vitrification and Nanowarming of Kidneys," *Advanced Science,* vol. 8, no. 19, Oct 2021, Art no. 2101691, doi: 10.1002/advs.202101691.

[3] O. J. Oziri *et al.*, "Scalable Purification of Iron Oxide Nanoparticles for Organ Cryopreservation and Transplantation," *Small : nano micro.,* 2025, doi: 10.1002/smll.202504910.

[4] Z. Gao *et al.*, "Vitrification and Rewarming of Magnetic Nanoparticle-Loaded Rat Hearts," *Advanced Materials Technologies,* vol. 7, no. 3, Mar 2022, Art no. 2100873, doi: 10.1002/admt.202100873.

[5] A. Chiu-Lam, E. Staples, C. J. Pepine, and C. Rinaldi, "Perfusion, Cryopreservation, and Nanowarming of Whole Hearts using Colloidally Stable Magnetic Cryopreservation Agent Solutions," *Science Advances,* vol. 7, no. 2, Jan 2021, Art no. eabe3005, doi: 10.1126/sciadv.abe3005.

[6] D. Potter, A. Jarrah, T. Sakai, J. Harrah, and M. Holliday, "Character of Function and Size in Kidney during Normal Growth of Rats," *Pediatric Research,* vol. 3, no. 1, pp. 51-&, 1969, doi: 10.1203/00006450-196901000-00007.

[7] J. Lee, J. Taylor, and S. Downing, "Comparison of Ventricular Weights and Geometry in Newborn, Young, and Adult Mammals," *Journal of Applied Physiology,* vol. 38, no. 1, pp. 147-150, 1975, doi: 10.1152/jappl.1975.38.1.147.
